# Enhancing Biomolecule Analysis and 2DMS Experiments by Implementation of (Activated Ion) 193 nm UVPD on a FT-ICR Mass Spectrometer

**DOI:** 10.1021/acs.analchem.2c02354

**Published:** 2022-11-01

**Authors:** Alina Theisen, Christopher A. Wootton, Anisha Haris, Tomos E. Morgan, Yuko P. Y. Lam, Mark P. Barrow, Peter B. O’Connor

**Affiliations:** Department of Chemistry, University of Warwick, Coventry CV4 7AL, U.K.

## Abstract

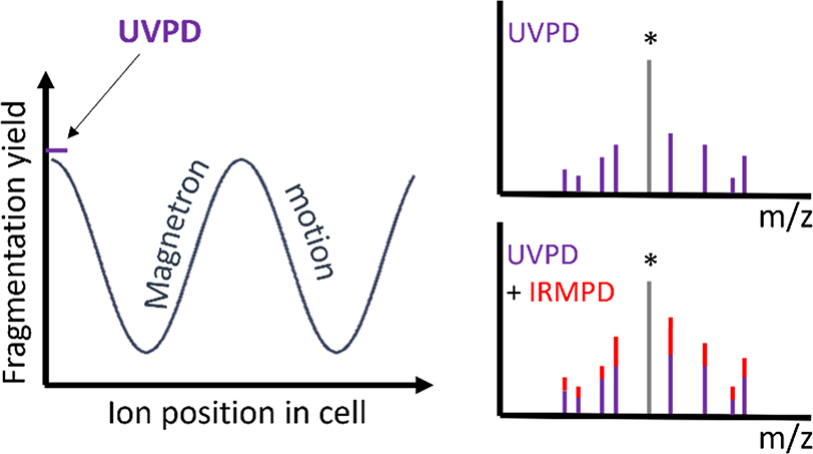

Ultraviolet photodissociation is a fast, photon-mediated fragmentation method that yields high sequence coverage and informative cleavages of biomolecules. In this work, 193 nm UVPD was coupled with a 12 Tesla FT-ICR mass spectrometer and 10.6 μm infrared multi-photon dissociation to provide gentle slow-heating of UV-irradiated ions. No internal instrument hardware modifications were required. Adjusting the timing of laser pulses to the ion motion within the ICR cell provided consistent fragmentation yield shot-to-shot and may also be used to monitor ion positions within the ICR cell. Single-pulse UVPD of the native-like 5+ charge state of ubiquitin resulted in 86.6% cleavage coverage. Additionally, IR activation post UVPD doubled the overall fragmentation yield and boosted the intensity of UVPD-specific x-type fragments up to 4-fold. This increased yield effect was also observed for the 6+ charge state of ubiquitin, albeit less pronounced. This indicates that gentle slow-heating serves to sever tethered fragments originating from non-covalently linked compact structures and makes activation post UVPD an attractive option to boost fragmentation efficiency for top-down studies. Lastly, UVPD was implemented and optimized as a fragmentation method for 2DMS, a data-independent acquisition method. UVPD-2DMS was demonstrated to be a viable method using BSA digest peptides as a model system.

## Introduction

Ion activation methods are crucial to the tandem mass spectrometry analysis of all kinds of molecules and play a large part in most experiment designs involving mass spectrometry. A fragmentation method that has gained increasing popularity in recent years is ultraviolet photodissociation (UVPD), which has been largely pioneered by the Brodbelt group as a versatile tool to analyze a wide variety of molecules including lipids, sugars, proteins and non-covalent complexes.^1−6^ For the analysis of proteins, for instance, UVPD is able to inform on all aspects of structures from primary to secondary, tertiary, and even quarternary.^7^ UVPD using 193 nm photons has been shown to produce extensive cleavage coverage even for larger species, which other activation methods may struggle to achieve.^8,9^

UVPD fragmentation yield correlates well with B-factors in solution, that is, the flexibility of secondary structural elements, with flexible regions fragmenting more readily than rigid elements.^3^ Photodissociation at 193 nm is also able to reveal the *cis*/*trans* isomerization of proline residues.^10^ Perturbation of the native-like fold of compact protein ions is reflected in UVPD spectra, and statistical analysis of fragment abundances may aid the assessment of initial protein structures in experiments where ion mobility analysis is unavailable.^11,12^ The ability of UVPD to cleave covalent bonds while leaving non-covalent linkages intact makes it an ideal candidate for studying protein–ligand binding while simultaneously highlighting conformational changes that may occur on binding.^13−15^ This characteristic also lends itself to the analysis of post-translational modifications (PTMs) that may be labile and not retained using collision-induced dissociation.^1,16^

The modulation of laser pulse energy has been utilized to induce the subunit disassembly of large, non-covalent protein complexes varying between symmetrical and asymmetrical partitioning and gain insight on subunit connectivity and stoichiometry.^3,17^

While many of the mechanistic studies on 157 and 193 nm UVPD were performed on time-of-flight instruments and UVPD is currently implemented predominantly on Orbitraps, the early studies combining UV lasers and mass spectrometry were performed on FT-ICR instruments and eventually led to the discovery of ECD.^18−26^ More recently, Shaw, Robinson, and Paša-Tolić implemented 193 nm UVPD on a 15 T FT-ICR instrument with a seven-segment cell and demonstrated the switch between UVPD-type spectra and photo-ECD-type spectra by modulating the parameters affecting the generation of electrons on metal surfaces impinged by the laser beam.^27^

Absorption of a single photon in the vacuum UV range (<200 nm) may be sufficient to cause electronic excitation and protein backbone cleavage, resulting in a large variety of all fragment types (a/b/c/x/y/z) including hydrogen-rich (+1/+2) or hydrogen-deficient (−1/–2) species.^8,28^

The large variety of fragments obtained by UVPD has been hypothesized to be insufficiently explained by direct dissociation only, and it has been suggested that UVPD proceeds via a combination of direct and statistical dissociation,^29^ the degree at which each one occurs may also vary with wavelength.^30,31^ While slow-heating of 213 nm UVPD products of the 13+ charge state of ubiquitin did not result in an increase in UV-specific fragments in a study conducted by Halim et al.,^32^ the sensitivity of UVPD spectra to precursor conformational states for low, compact protein charge states as well as the overall low fragmentation yield may indicate that similar to ECD, non-covalently linked fragments may be present.^12^ However, in contrast to ECD, these would not be charge-reduced so they coincide with the precursor *m*/*z* and are effectively invisible until released by additional activation via slow heating methods.

UVPD of intact proteins produces complex spectra with an extensive range of diverse fragments, making FT-ICR an ideal partner due to its ultrahigh resolving power and mass accuracy. Due to the applicability of UVPD on a large variety of samples and especially its performance in protein top-down experiments, in this study, we implement 193 nm UVPD on a commercial 12 T Bruker solariX FT-ICR MS equipped with an infinity cell and IRMPD MS/MS without requiring modification to the main instrument. Further, we combine UVPD fragmentation with subsequent IRMPD activation to improve fragmentation yield and implement UVPD as a fragmentation method for two-dimensional MS analysis (2DMS).

## Experimental Section

Leucine enkephalin (LeuEnk) (58822-25-6), bovine ubiquitin (79586-22-4), bovine serum albumin (BSA) (9048-46-8), and ammonium acetate (631-61-8) were purchased from Sigma-Aldrich (UK). LCMS-grade methanol was purchased from VRW (USA), and formic acid was obtained from Honeywell Fluka (Germany). Ultrapure water was obtained from a Milli-Q Direct-Q UV3 water purification system (Merck Millipore, USA). Bovine serum albumin digest was prepared according to the protocol in the Supporting Information. LeuEnk was prepared in 50% methanol with 0.1% formic acid (FA) to a concentration of 5 μM. Ubiquitin was dissolved in 50 mM ammonium acetate to a final concentration of 5 μM.

### FT-ICR-MS Experiments

Samples were ionized in positive-ion mode using a nanoESI source built in-house with a typical capillary voltage of 0.5–0.8 kV. Nanospray emitters were pulled in-house on a Sutter P-97 Flaming/Brown-type filament tip puller (Sutter Instruments, Novato, USA) using thin-wall borosilicate glass capillaries with an inner diameter of 1 mm (TW120F-3, World Precision Instruments, USA). A nichrome wire was inserted into the back of the capillaries for electrical connection. Transients were acquired with 4 mega-word (2^22^, 32-bit) data points, and mass spectra were generated with between 10 and 100 summed transients with a detection mass range of 150–3000 *m*/*z*.

Data was analyzed using Bruker DataAnalysis 4.3 and OriginLab 2019. Peak lists were generated using the SNAP peak detection algorithm built into DataAnalysis. Fragments were assigned with a tolerance of 2 ppm using software written in-house (Cookson 3.0, included in the Supporting Information). A more detailed description of the fragment assignment criteria and the equation for the calculation of fragmentation yields can be found in the Supporting Information.

### Two-Dimensional Mass Spectrometry

2DMS spectra were acquired using a Gaumann pulse sequence as described previously for a solariX FT-ICR MS.^33^ In the 2DMS experiment herein, 8192 scans, each utilizing a different incremental delay *t*_1_ and consisting of a transient containing 1 MW data points (2^20^), were acquired. Data was initially processed using SPIKE,^34^ denoised with urQRd rank 15, and further analyzed using a program written in-house termed T2D.^35^

### Implementing UVPD with IRMPD on the FT-ICR

**Figure 1:**
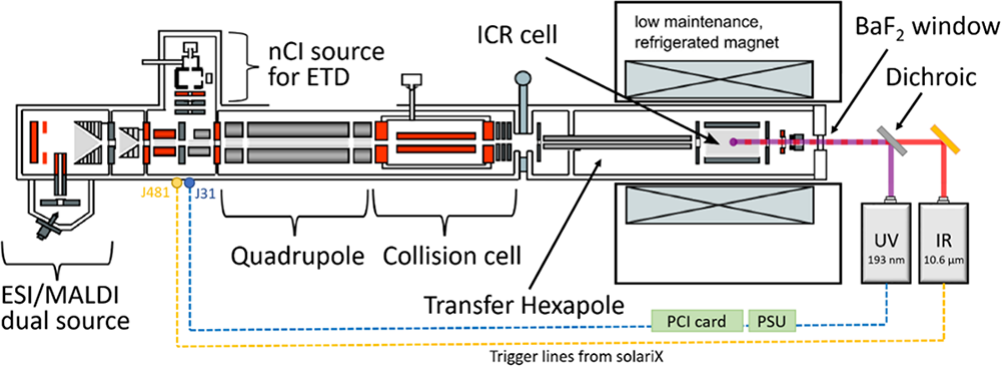
Schematic of the 12 T Bruker solariX setup with UVPD and IRMPD.

A 193 nm ArF excimer laser (ExciStar XS 500, Coherent) was introduced into the infinity cell of a commercial 12 T Bruker solariX (Bruker, Bremen, Germany) with IRMPD capabilities. Both beams were co-aligned using a custom-coated dichroic mirror (Lambda Research Optics, US), and no hardware modifications of the commercial instrument itself were required. The full description of the setup (Figure 1) including optics, a wiring diagram, a triggering circuit, and pulse programs can be found in the Supporting Information.

## Results and Discussion

Leucine enkephalin (LeuEnk) was utilized as a model peptide to test UVPD performance of the system. The singly charged ion was isolated in the quadrupole and subjected to a varying number of laser shots with varying pulse energies immediately after entering the FT-ICR cell. After a user-defined delay of 120 ms (to allow for a variable number of shots), ions were excited and detected as usual. The resulting UVPD spectrum acquired with a single laser shot at 2 mJ can be seen in SI Figure S1A. While the most intense ions are a-type ions, a large variety of fragments were produced, providing a cleavage coverage of 100,% including cleaving the tyrosine side chain. Internal fragments and loss of NH_3_ were also observed. With a single laser pulse at 2 mJ, a fragmentation efficiency of 5.5% was achieved. This increased linearly with an increased number of laser shots (*R*^2^ = 0.99; see SI Figure S1B) and an increase in pulse energy (*R*^2^ = 0.97; see SI Figure S1C), indicating a single-photon process in which one photon causes one fragmentation event.^36^

### Mapping Magnetron Motion in the Infinity Cell Using UVPD

Due to being exposed to both electric and magnetic fields, ions in ICR cells exhibit three types of motion: magnetron and cyclotron motion in the *xy* plane and trapping motion along the *z* axis.^37^ Previous studies have shown that best results are achieved when fragmentation events are timed to ion motion to maximize the overlap between the ion cloud and the fragmentation zone.^27,38,39^ Trapping motion and pre-excitation cyclotron motion are negligible in the context of ion–fragmentation zone overlap.

**Figure 2:**
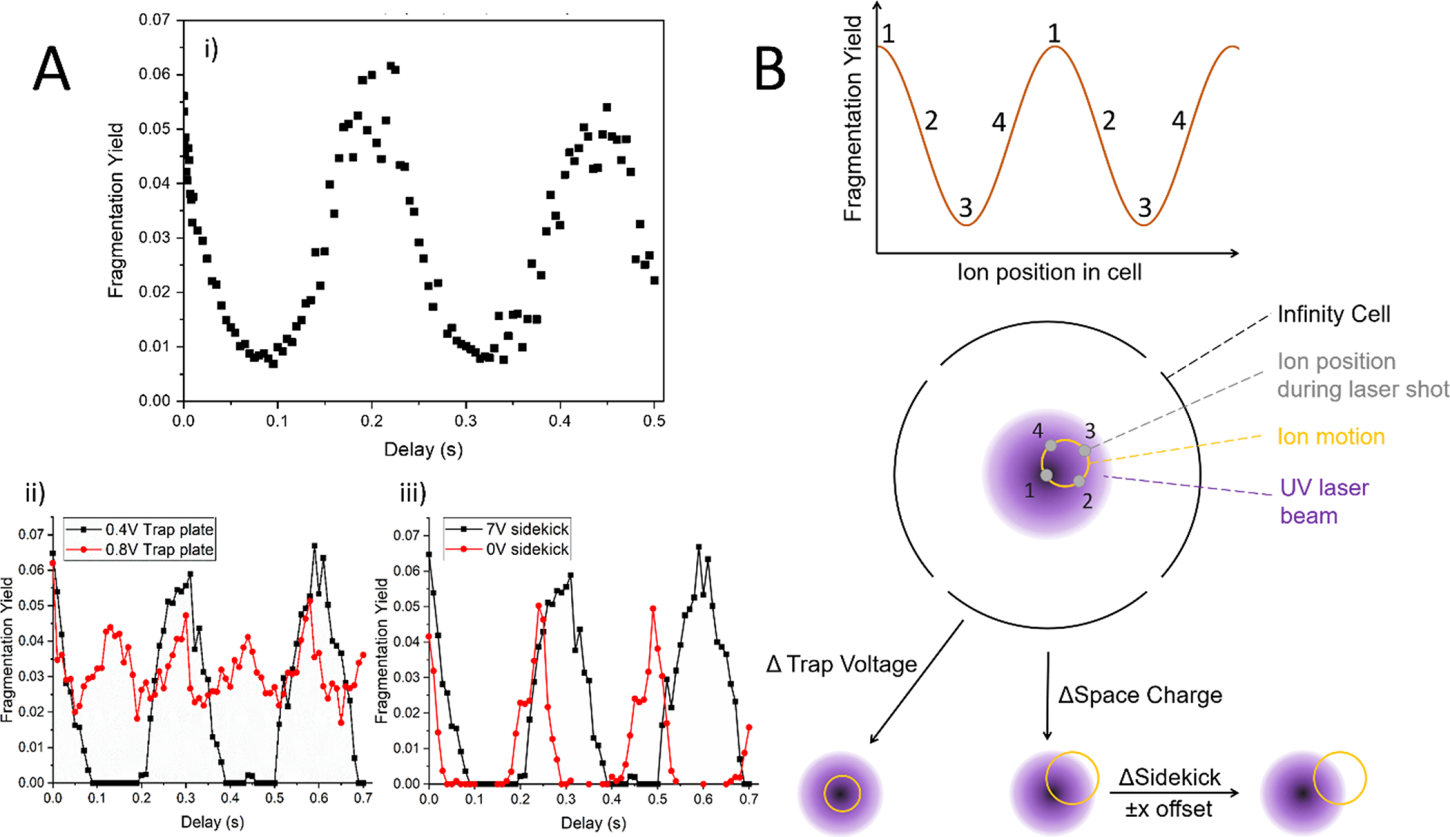
Mapping ion motion in the infinity cell using UVPD of LeuEnk. (A) Fragmentation yield as a function of the delay between ions entering the infinity cell and the laser being fired. A sinusoidal relationship is observed as ions move in and out of the center of the laser beam. The three plots show the variation in ion motion in relation to the laser beam when various parameters are changed. (B) Schematic representation of the changes in ion motion and center of gyration as parameters such as space charge, trap voltage, and lens offset were adjusted. Changes shown summarize data collected only. Drawing is for visual representation and not drawn to scale (real diameters: infinity cell, 6 cm; laser beam, 2.5 mm).

Magnetron motion is a periodic motion centered on the electric field center of the ICR cell (and not necessarily the magnetic center that induces cyclotron motion), which follows the equipotential contours of the cell and starts as ions enter the ICR cell. If the center of magnetron gyration is not the center of cyclotron gyration, then the magnetron motion modulates ions radially and, thus, within the fragmentation region, as time goes on. Combined with a fast probe event (such as a UVPD laser pulse), these effects will change the resulting fragmentation yield observed (see Figure 2B). The laser beam is brightest in the center and decreases in energy with increasing radius with roughly a top-hat profile because of the clipping of the laser as it passes the hollow-cathode electron gun; therefore, ions also experience differing amounts of fragmentation if they move in/out of the fragmentation zone, further modulating the resulting yield with respect to time.

Inserting and incrementing a delay between ions entering the FT-ICR cell and the laser shot allows visualization of the ion motion (Figure 2Ai). On the timescale used, ions start in the center of the cell so that a fragmentation maximum is observed at a delay of 0, which then decreases as ions move outward radially. After ∼200 ms, ions returned to their initial radial position as evidenced by the sine wave maxima; this motion is periodic and representative of the magnetron motion. Not only does this method allow tracking of the frequency of magnetron motion, but because the diameter of the laser beam (2.5 mm) is known because of the 2.5 mm orifice of the electron gun, estimation of the dimension of the ion motion can be made. If the fragmentation yield drops to 0, the radial motion exceeded the boundaries of the laser beam, and therefore, ions moved out of the center by more than 1.25 mm. Note that this argument assumes that the cell is well aligned with the magnetic field so that the ion packets are not moving in and out of the fragmentation zone as they oscillate axially.

Jertz and co-workers previously used an incremental delay after ion trapping to measure magnetron motion via harmonic signals and termed this methodology a post-capture delay (PCD) curve.^40^ In these experiments, which were performed in a dynamically harmonized ICR cell also referred to as the “ParaCell”, the intensity of the second harmonic cluster was monitored as a function of the delay between ion trapping and ion excitation. As ions move off-axis due to magnetron motion, the center of their excited cyclotron radii will also be off-axis, resulting in a stronger image current on the detection electrode toward which the center of gyration is offset. This results in more intense second harmonic signals after Fourier-transform, with increasing off-axis center of cyclotron motion.

In order to compare and validate the UVPD magnetron motion curve obtained on the infinity cell to the established PCD methodology in the ParaCell, the intensity of the second harmonic was plotted for each delay (see SI Figure S2). Two things should be noted when comparing the two curves directly: (1) The maxima of the laser curve represent the innermost ion position, whereas the maxima of the PCD curve represent the outermost ion position in relation to the cell center; hence, both curves are inverted with respect to each other. (2) Due to a delay (120 ms) between the laser shot and ion excitation, the PCD curve probes the ion position at a slightly later point in time than the UVPD event, resulting in an offset between the two curves. As expected, the frequency and shape of both the laser PCD and traditional PCD curves are identical, highlighting that both methods probe the same ion motion and enable the optimization of UVPD fragmentation to be as reproducible as possible shot-to-shot.

Space charge (i.e., number of ions/charges in the cell), the user-defined trap plate voltages, and the so-called “sidekick” voltage ions experience upon entering the cell influence the center and radius of magnetron motion. Doubling the trapping voltage from 0.4 V (Figure 2Aii, black line) to 0.8 V (Figure 2Aii, red line) results in a doubling of the measured magnetron frequency. While the maxima are lower at 0.8 than 0.4 V, the minima are much higher, and therefore, increasing the trapping voltage produced a more consistent fragmentation yield throughout in this example. This represents the higher trapping voltage moving the center of magnetron motion toward the center of the laser beam and ICR cell (Figure 2B). Ions then orbit around the brightest part of the beam rather than crossing it, resulting in lower maxima, but the ions also stay within a similar brightness of the laser fragmentation zone, producing more consistent shot-to-shot fragmentation. The PCD curves measured (see SI Figure S3) correlate well with the observed fragmentation yields in Figure 2; higher trapping voltage resulted in lower second harmonics and doubled frequency magnetron motion recorded for the parameters tested.

Detuning the ions’ cell entry point from centered to off-axis using the sidekick voltage also results in lower fragmentation maxima (Figure 2Aiii), suggesting that ions no longer passed through the center of the beam under these conditions. Fragmentation yield also drops to 0 between maxima as ions left the fragmentation zone entirely at these time points. The corresponding PCD curves can be found in SI Figure S4.

While frequency varies between the tested conditions, a delay of 0 ms consistently produced a fragmentation yield maximum and represents a simple target for UVPD experiments utilizing a single laser shot. Consequently, this was chosen for further experiments. For faster-repetition rate lasers such as 500 Hz systems (including the ExciStar XS used here), multiple laser shots are still viable with an initial delay of 0 ms. Up to five shots could be fired during the first 10 ms of ion motion before fragmentation yield dropped significantly by more than 15%.

However, the timing of laser pulses could also be tailored to subsequent local maxima found within the curve, allowing maximum fragmentation efficiency per laser pulse when multiple laser pulses are desired. While this approach would be more time-intensive as there is a longer wait period in between laser shots, this would provide the best fragmentation yield and may be beneficial for discontinuous ion sources such as DAPI^41^/MALDI to get the most fragmentation yield from each packet of ions as well as systems that employ lasers with slower repetition rates.

The results presented herein suggest that UVPD in FT-ICR may be particularly suitable for any system where magnetron motion can be minimized and ions can be centered on the cell axis through careful tuning of cell parameters.

### Native Top-Down Combining UVPD with Gentle Post-Activation by IRMPD

**Figure 3:**
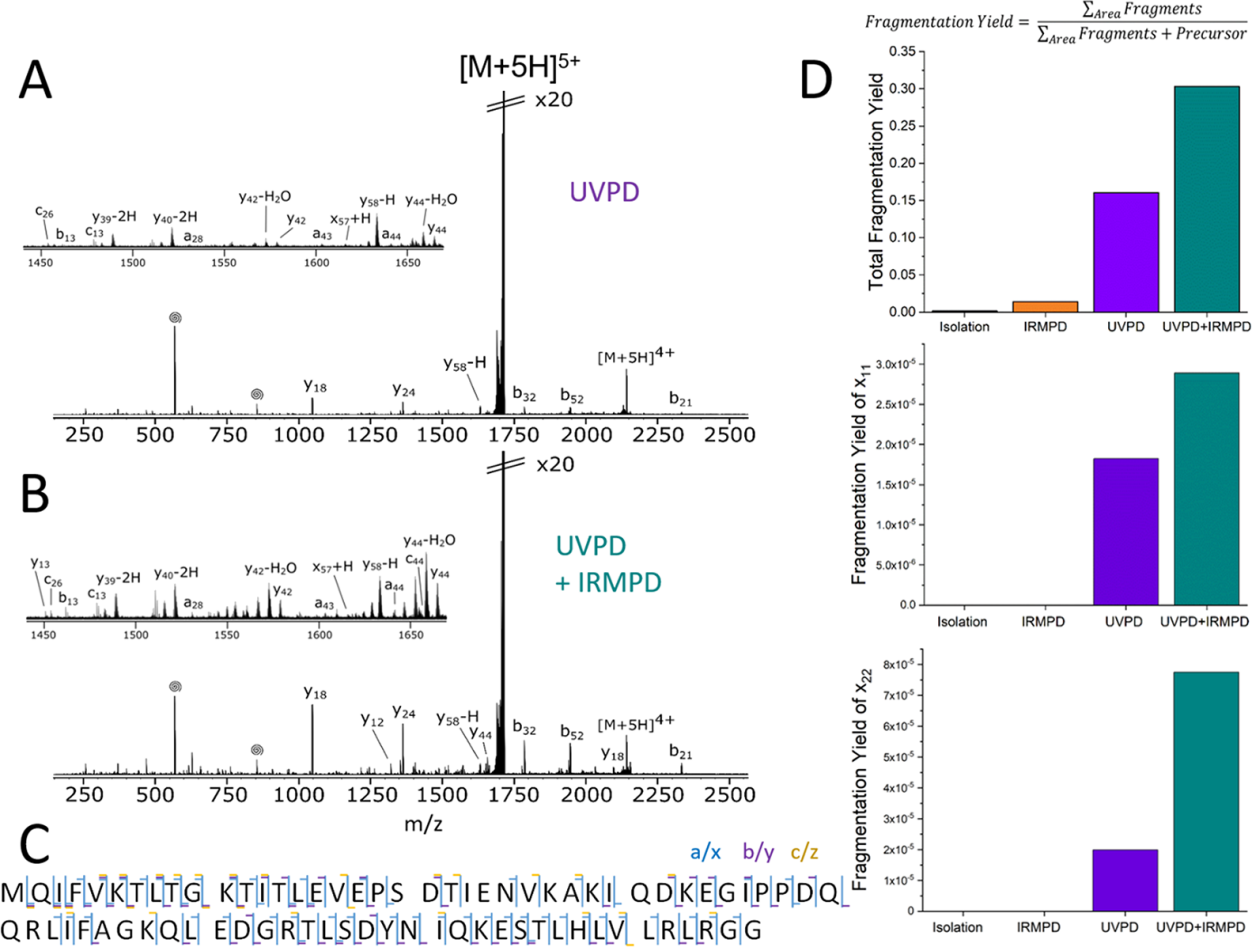
193 nm UVPD of 5+ ubiquitin with and without subsequent IR activation. (A) 193 nm UVPD spectrum of [M + 5H]^5+^ produced using one laser pulse at 7 mJ. (B) Spectrum resulting from 110 ms IR activation after UVPD. (C) Cleavage map with all identified fragments corresponding to 86.6% cleavage coverage. (D) Comparison of fragmentation yields between various experimental modes. IR activation of UV products results in an increase in fragmentation yield overall as well as for UVPD-specific x-ions.

UVPD MS/MS is particularly relevant for protein analysis due to its high cleavage coverage^8^ and ability to locate PTMs^1^ and dissociate the protein backbone without causing large-scale protein unfolding, allowing native MS/MS studies.^12^Figure 3A shows MS/MS results from ions isolated from ubiquitin solution analyzed by native MS in 50 mM ammonium acetate. The 5+ charge state was isolated in the quadrupole, and ions were subjected to a single UV laser pulse at 7 mJ in the ICR cell. The resulting UVPD spectrum can be seen in Figure 3A. A wide variety of fragments were observed including a, a + 1, a + 2, c, x, x + 1, y, y – 1, y – 2 and z types, producing an overall cleavage coverage of 86.6% (Figure 3C). The most abundant fragment types in terms of intensity were the y-type ions, closely followed by a and b (see SI Figure S5). The most intense fragments observed (y_18_, y_24_, b_32_, b_52_, b_21_, labeled on Figure 3A) arise from cleavage of the C-terminal side of aspartic acid.

Previous studies using UVPD on ubiquitin 5+ showed high intensity a-ions, followed by x- and y-type ions as the more intense species detected and hypothesized a “non-specific fragmentation” favorability.^8^ These results, acquired on an Orbitrap Elite platform, were obtained in a very different UVPD MS/MS environment compared to the results herein, and these differences between instrument platforms afford some explanation to the differences observed in the results. The ICR cell used here was operated at a vacuum of 3 × 10^–10^ mbar, while the HCD cell of the Orbitrap, with a collision gas pressure of 5 mTorr (∼6 × 10^–3^ mbar), was much higher in pressure.^8,42^ UVPD fragmentation is generally thought to be a result of a combination of fast dissociation from electronic excited states, slower fragmentation from remaining ground-state radical rearrangements similar to ECD, and slow dissociation from the ground state after intramolecular vibrational energy redistribution (IVR), which would result in CID-like fragmentation.^8,29^ The complete absence of collisional cooling during UVPD on the FT-ICR is therefore likely to be a factor in the observed fragmentation patterns.^43^ Another factor is the absence of additional activation post UVPD in the FT-ICR cell unless specifically introduced (e.g., via IRMPD or SORI-CID), whereas on other platforms, such as the Orbitrap, UVPD is not conducted inside the high-vacuum region of the Orbitrap detector and instead in a separate low-vacuum linear ion trap; ions have to travel from that linear ion trap to the Orbitrap analyzer post UVPD, which involves traversing ion optics that can cause further collisional activation and changes in the observed fragments.

While the overall fragmentation yield of 0.16 (i.e., 16%) was sufficient for the 5+ ubiquitin species to obtain a cleavage coverage of 86.6%, improvements in yield and, therefore, the signal-to-noise ratio of low-intensity species may aid UVPD analysis. Similar to what is known from ECD, tethered fragments held together by non-covalent intramolecular bonds may be present after UVPD, which are coincident with the precursor *m*/*z*.^12,44,45^ The method of 213 nm UVPD has previously been coupled with CID^11^ or IRMPD^32^ for the post-activation of UV products, which improved fragmentation yield for compact protein species, while this effect was less pronounced or absent for more extended or denatured conformations.

Here, 10.6 μm IR activation was implemented after 193 nm UVPD MS/MS. After a user-defined wait period between the triggers for UVPD and IRMPD (120 ms), ions within the ICR cell, which also includes the UVPD photoproducts, were irradiated with the IR laser for 110 ms (Figure 3B). The IRMPD irradiation time was chosen as the maximum value at which IRMPD itself, without preceding UVPD, did not produce detectable backbone cleavage (see control spectra in SI Figure S6). Gentle post-activation of UVPD products resulted in a drastic increase of the overall fragmentation yield from 0.16 to 0.3, almost doubling the overall fragment intensity. The increase in fragmentation yield was also observed for UVPD-specific fragments (see Figure 3D), which cannot originate from protein fragmentation due to slow-heating. This was particularly pronounced for fragment x_22_ with a four-fold increase in intensity over UVPD alone. Therefore, the increase in fragmentation yield is predominantly due to the release of tethered fragments produced by UVPD of compact protein conformations, in close correlation with results shown for IR-ECD.^44,45^ It is not possible to fully exclude the possibility of secondary fragmentation due to IRMPD of radical species produced by UVPD; however, no new fragments were observed for UVPD-IRMPD of ubiquitin, only an increase in intensity of previously detected fragmentation channels, so any radical/secondary fragmentation may only have created non-unique fragments already observed in the pure UVPD spectrum, though this may change for other species of interest.

The 6+ charge state of ubiquitin was also studied using IR-activated UVPD (see SI Figure S7). UVPD itself produced cleavage coverage of 84% and an overall fragmentation yield of 0.12. An increase in IR irradiation time post UVPD correlated with an increase in fragmentation yield up to an irradiation time of 120 ms, after which IRMPD itself was sufficient to cause fragmentation without preceding UVPD (SI Figures S7D and S8). Post-activation of UV photoproducts increased the observed overall fragmentation yield by almost 50% up to 0.17; despite being a lower increase than for the 5+ charge state, this still represents a drastic improvement and highlights the potential of activated ion UVPD for top-down proteomics, especially for native species which are often observed in lower, thus less MS/MS favorable, charge states. While the UVPD MS/MS of activated, extended conformations of the 5+ and 6+ charge states of ubiquitin have previously been shown to produce higher fragmentation yields in part due to removing the intramolecular bonds that otherwise may tether fragments,^12^ preactivation of ions using 13 V collisional activation prior to UVPD did not significantly improve fragmentation yield in this setup (SI Figure S7E). CID occurs in the collision cell, where ions are accumulated and stored for up to 1 s before transfer to the ICR cell, which may be sufficient time for cooling, refolding, or structural rearrangement to occur.^46,47^

### Implementation and Optimization of UVPD-2DMS

**Figure 4:**
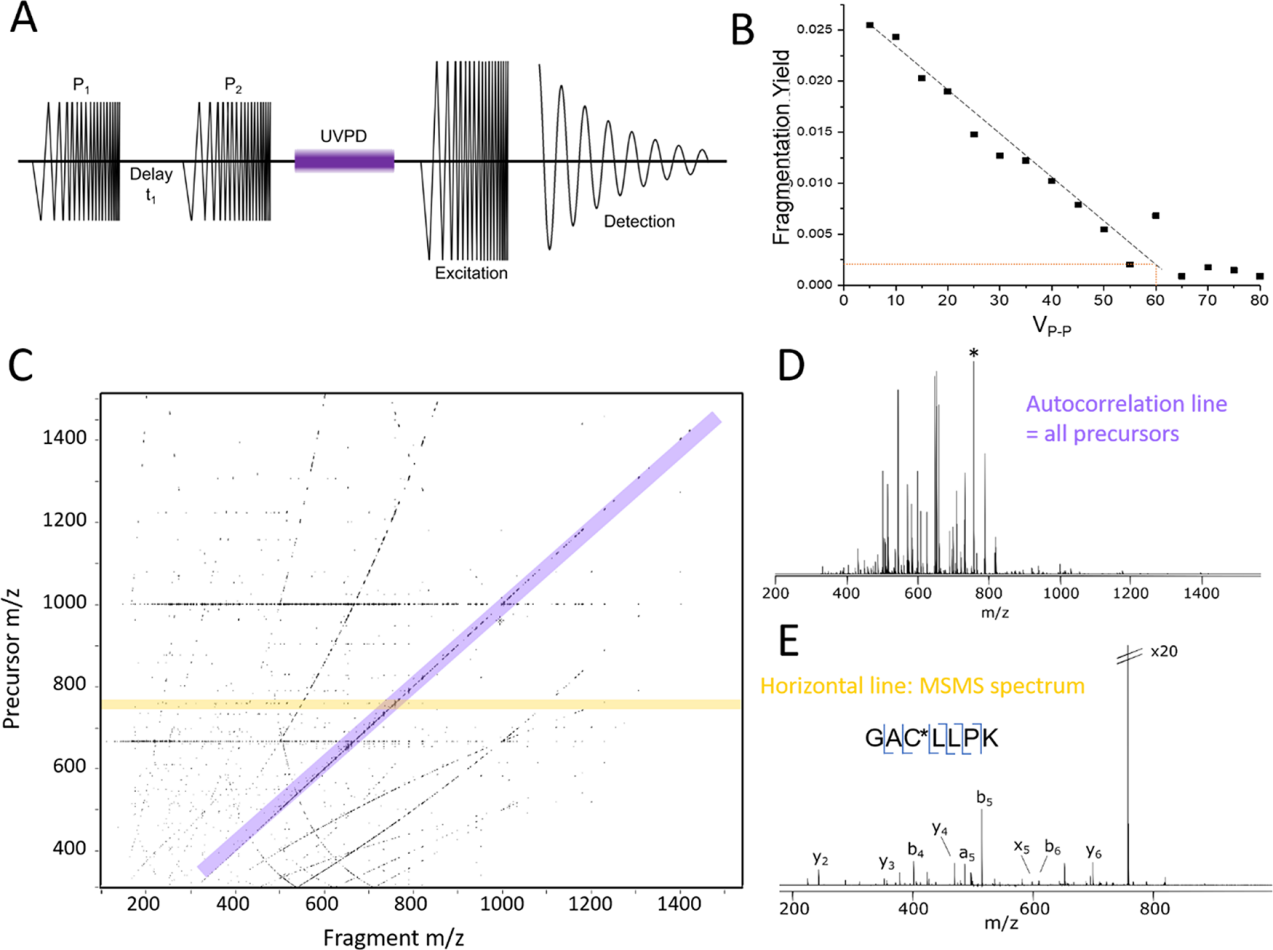
UVPD-2DMS of BSA digest. (A) Schematic of the pulse–delay–pulse sequence used for 2DMS experiments. Increments in delay *t*_1_ result in ions occupying different radii in each spectrum, resulting in varying fragmentation yields. (B) UVPD optimization curve for 2DMS experiments. Sweep excitation (*V*_P-P_) is proportional to the ion’s radius in the cell. The minimum fragmentation efficiency chosen for 2DMS, ∼10% of the fragmentation maximum, is represented by the orange line and corresponds to a *V*_P-P_ of 60 V, which is equal to a power level of 30 V used for both excitation pulses P_1_ and P_2_. (C) 2DMS contour plot of BSA digest obtained using one laser pulse at 7 mJ pulse energy. Each “dot” observed at this zoom level is a three-dimensional peak. (D) Autocorrelation line where all precursors that fragment in the experiment are shown. The horizontal fragmentation line for the peptide marked with an asterisk is shown in (E).

Two-dimensional MS (2DMS) is a data-independent acquisition method where all analytes observed in a sample are fragmented and analyzed simultaneously.^33^ The basis of this technique is a simple pulse–delay–pulse sequence (Figure 4A), which modulates ion positions radially depending on their *m*/*z*, and a spatially defined fragmentation zone. The first excitation pulse P_1_ induces phase coherence and excites ions to a higher radius. During the incremental delay *t*_1_, ions acquire phase, i.e., they separate in space along the orbit according to their *m*/*z*.^48^ Depending on their radial position (thus phase) at excitation pulse P_2_ in relation to the excitation electrodes, ions are then either excited to an even higher radius or de-excited back toward the cell center, which coincides with the center of the laser beam.

Fragment ion intensities therefore increase and decrease at the same frequency at which their precursor is moving in and out of the fragmentation zone. Thus, the FFT of this data provides very precise correlation of fragments to their precursor ions despite all precursors fragmenting in all scans. The result of the 2DMS experiment is displayed as a three-dimensional contour plot where the *x* axis represents the precursor *m*/*z*, the *y* axis represents the fragment *m*/*z* and the *z* axis represents the peak intensity.

It is important that the maximum radius that ions are excited to is kept within the outer boundary of the fragmentation zone to ensure that the fragment ion intensity does not drop to 0 at any point in the 2DMS experiment as this would produce a clipped sine wave and introduce artifacts during FFT.^49^ In order to implement UVPD as a fragmentation method for 2DMS, the fragmentation zone was mapped and the power level of the excitation pulses were optimized to both keep ions within the fragmentation zone but also maximize the amplitude of ion position modulation. The optimization plot for UVPD-2DMS can be seen in Figure 4B, where fragmentation yield is plotted as a function of excitation power (in *V*_P-P_), which correlates to ion position. At low excitation power, ions stay close to the center of the fragmentation zone and move to a higher end radius as power increases. The *V*_P-P_ value of 60 was chosen as the optimized power level, corresponding to slightly less than 10% of the maximum fragmentation efficiency as a lower point. Since 2DMS uses two excitation pulses, this translates to a power level of 30 *V*_P-P_ for each pulse, P_1_ and P_2_.

The first UVPD-2DMS spectrum of the BSA digest is displayed in Figure 4C. Precursors, that is, all ions that fragment during the 2DMS experiment, align along the autocorrelation line where *x* = *y* (Figure 4D). An example horizontal MSMS line extracted for the peptide GAC*LLPK (where * indicates an alkylation event), the most intense precursor on the autocorrelation line, is shown in Figure 4E, where full cleavage coverage was achieved. These results clearly show the viability of UVPD as a fragmentation method for 2DMS with the BSA digest spectrum serving as proof of principle.

As the fragmentation efficiency of UVPD is low without post-activation, improvements in the signal-to-noise ratio may be needed to detect very low-intensity species. The next developments in UVPD-2DMS will include incorporating the IRMPD post-activation established herein into the 2DMS method to increase fragment intensity. Adaptations to the pulse program can also be made to allow more than one scan per incremental delay to be acquired. Overall, these improvements should not only improve efficiency of UVPD-2DMS for bottom-up analysis but also improve its performance for top-down measurements.

## Conclusions

Herein, the successful implementation of 193 nm UVPD on a commercial 12 T Bruker solariX instrument is shown while retaining IRMPD and ECD capabilities via incorporation of an external, custom-made dichroic mirror enabling IR transmission and UV reflectance. Monitoring the UV fragmentation yield as a function of the delay after ions enter the infinity cell allowed the acquisition of a laser-PCD curve, visualizing magnetron motion of ions within the cell, which provides metrics for the optimization of timing to produce more linear shot-to-shot response for UVPD MS/MS. High sequence coverage was obtained for low charge states of ubiquitin, with fragments of all types (a/a + 1/b/c/x/x + 1/y/y – 1/z) providing comprehensive analysis of the species of interest. Gentle IR activation of UV photoproducts was able to double the fragmentation yield for the low charge states of ubiquitin and improve the signal-to-noise ratio for low-abundance fragments. This result is particularly promising for application to native top-down experiments. Further, UVPD as a fragmentation method for 2DMS was implemented and optimized, where we envision incorporation of IR activation will also be of great benefit in future.

The ability to conduct such combinations of dissociation experiments on commercial equipment can enhance the fragmentation yield of UVPD experiments and may extend the range of systems that can be effectively studied, in a similar fashion to AI-ECD and AI-ETD experiments, which have been shown to be so effective at both top-down and bottom-up analyses.^44^

## Supporting Information Available

Detail of experimental methods, additional experimental data, solariX pulse programs, and other software used in this work (PDF)
