# Supplementary material for: Enhancing Biomolecule Analysis and 2DMS Experiments by Implementation of (Activated Ion) 193 nm UVPD on a FT-ICR Mass Spectrometer
Source: Anal Chem. 2022 Nov 1;94(45):15631–8. doi: 10.1021/acs.analchem.2c02354 (PMC9670024; doi:10.1021/acs.analchem.2c02354)
Supplement: Supplementary file 1 — ac2c02354_si_001.pdf [file ac2c02354_si_001.pdf]

Enhancing biomolecule analysis and 2DMS experiments by implementation of (activated ion-) 193 nm UVPD on a FT-ICR mass spectrometer

Supplementary Information

Alina Theisen, Christopher A. Wootton, Anisha Haris, Tomos E. Morgan, Yuko Lam, Mark P. Barrow, Peter B. O'Connor

Department of Chemistry, University of Warwick, Coventry, CV4 7AL, UK

Table of contents

SI Methods 2

Preparation of bovine serum albumin digest 2

Implementing UVPD with IRMPD on the FT-ICR 2

Fragment assignment and yield calculation 3

SI Figures 4

SI Figure 1: 193 nm UVPD of LeuEnk 4

SI Figure 2: Intensity of the 2nd harmonic as a function of the delay between ions entering the cell and the laser being fired 5

SI Figure 3: Intensity of the 2nd harmonic as a function of UVPD delay compared between low and high trap voltage settings 6

SI Figure 4: Intensity of the 2nd harmonic as a function of UVPD delay compared between a sidekick voltage of 0 V and 7 V 7

SI Figure 5: Fragmentation yield of 193 nm UVPD of $[M+5H]^{5+}$ by fragment type 8

SI Figure 6: Isolation only (top) and IRMPD control spectum (bottom) of $[M+5H]^{5+}$ Ubiquitin 9

SI Figure 7: 193 nm UVPD of 6+ Ubiquitin with and without subsequent IR activation as well as pre-activation in the collision cell 10

SI Figure 8: Isolation only (top), IRMPD 120 ms (middle) and CID 13 V (bottom) control spectra of $[M+6H]^{6+}$ Ubiquitin 11

SI Schematics 12

SI schematic 1: Wiring diagram of FET switch used for laser triggering 12

SI solariX pulse pograms used in this work 13

Standard UVPD experiments 13

UVPD followed by IRMPD experiments 20

UVPD-2DMS experiments, default properties 28

2DMS – UVPD experiments, master properties 45

SI Programs 47

Cookson 3.0 Fragment analysis 47

LaserControl software .... **Error! Bookmark not defined.**

SI Methods

Preparation of bovine serum albumin digest

Bovine serum albumin (BSA) was dissolved in 100 mM ammonium bicarbonate to give a protein concentration of 1 mg/ml. 3 µl of 50 mM dithiothreitol (DTT) was added to 50 µl of BSA and incubated for 30 minutes at 60° C to reduce disulphide bonds. To alkylate the sample, 3 µl of 100 mM iodoacetamide (IAA) was added to the solution and incubated, in the dark, at room temperature for 60 minutes. Next, 2 µl of bovine trypsin were pipetted into the BSA solution for digestion and 100 mM ammonium bicarbonate added to give a protein concentration of 0.5 mg/ml. A final incubation was carried out at 37° C for 16 hours. For purification, 1 mg of digested BSA sample was loaded onto a C18 reversed-phase solid phase extraction (RP-SPE) cartridge (Thermo Fisher Scientific, UK) and washed twice with 1 ml of 0.1% FA in H_2O to remove salts, excess DTT and IAA. Peptides were eluted with 1.5 ml of 20:80 H_2O:ACN solution containing 0.1% FA and dried down using a Savant SPD121D SpeedVac concentrator (Thermo Fisher Scientific, UK) prior to re-dissolving in H_2O to a final peptide concentration of 1 µg/µl.

Implementing UVPD with IRMPD on the FT-ICR

A commercial 12 T Bruker solariX (Bruker, Bremen, Germany) equipped with an infinity cell and IRMPD capability was used in this study. A 193 nm ArF excimer laser (ExciStar XS 500, Coherent) was co-aligned to the IR beam path (CO_2, 10.6 µm, Synrad J48-2, 25W) using a UV mirror optimised for 193 nm transmission (#47-982, Edmund Optics, >97% reflectance @ 193 nm) and a custom coated dichroic mirror that reflects UV and transmits IR (Lambda Research Optics, US) with an efficiency of 60% for both wavelengths. Optical access to the infinity cell was granted by a BaF_2 window which transmits 50% of the 193 nm photons. To enter the cell, both beams pass through the ECD hollow cathode which clips the beams down to a diameter of 2.5 mm. No adverse effects are observed on the ECD cathode.

Furthermore, as the infinity cell has inlet and exit orifices of 5 mm diameter, the 2.5 mm diameter laser beam is unlikely to contact any surfaces within the cell thus eliminating the risk of photoelectron production and photo-ECD events.

Geographical and safety constraints for the 193 nm laser dictated a ~2 m beam path in air, resulting in a further 50% power loss purely from air up to the instrument entry point. Considering all optics, beam divergence, beam path and clipping, around 5% of the laser power (measured at the laser exit) enters the infinity cell central volume which was sufficient for UVPD, and the effective beam profile inside the ICR cell is a near top-hat distribution. Overall, no hardware modifications of the instrument were required, purely the external laser setup (Figure 1).

In order to utilise both UVPD and IRMPD, separate triggers are required for both lasers. The IR laser was triggered by pin J481 on the solariX NICE electronics as installed by Bruker and was not modified.

Pin 35 on the AUX interface J31 at the front of the instrument was chosen as the trigger out for the UV laser and a positive TTL was incorporated at the appropriate point in the pulse program (included in the supporting information). The trigger first goes to a NI PCI card (PCI-6221, National Instruments) driven by a LabView program written in-house which allows the user to define the

number of laser shots, repetition rate, and timing of the UV shots after the trigger from solariX is received (link to LaserControl software can be found in SI Programs). The program ensures the appropriate amount, shape and timing (for this UV laser up to a repetition rate of 500 Hz) of the outgoing trigger pulse(s) which is then routed through a FET (circuit diagram included in SI Schematics) using an EL301R laboratory power supply (Aim-TTi, UK) to achieve the required current of 100 mA to trigger the excimer laser. The overall delay between pin 35 going high and a laser shot fired is 76.25 ± 2.5 µs as measured between trig to laser sync out by oscilloscope, and delay between sync out and laser pulse as described in the UV laser manual as 7 µs.

Fragment assignment and yield calculation

Fragments were assigned with a tolerance of 2 ppm using software written in-house (Cookson 3.0, included in the SI Programs) which checks for a, a+1, a+2, b, c, c·, x, x+1, y, y-1, y-2, z and z· fragments. Losses of -NH_3 and -H_2O were also considered for all fragment types. All fragments identified by the software were double checked manually. Internal fragments of ubiquitin were not assigned but counted towards the total fragmentation yield.

The fragmentation yield of LeuEnk was calculated according to the following relationship:

$$Fragmentation\,Yield = \frac{\sum_{intensity} Fragments}{\sum_{intensity} Fragments + \ Precursor}$$

For ubiquitin, the sum of the area was used rather than the sum of intensities as fragment ions were spread over a wider m/z range resulting in a larger disparity in resolution between fragments which would affect comparison of fragmentation e.g. between different charge states of ubiquitin. The same sequence fragment might be found at a different m/z (thus a different resolution), hence the intensity would be different, but the peak area remains proportional to the amount of ions detected, producing a more accurate comparison.

SI Figures

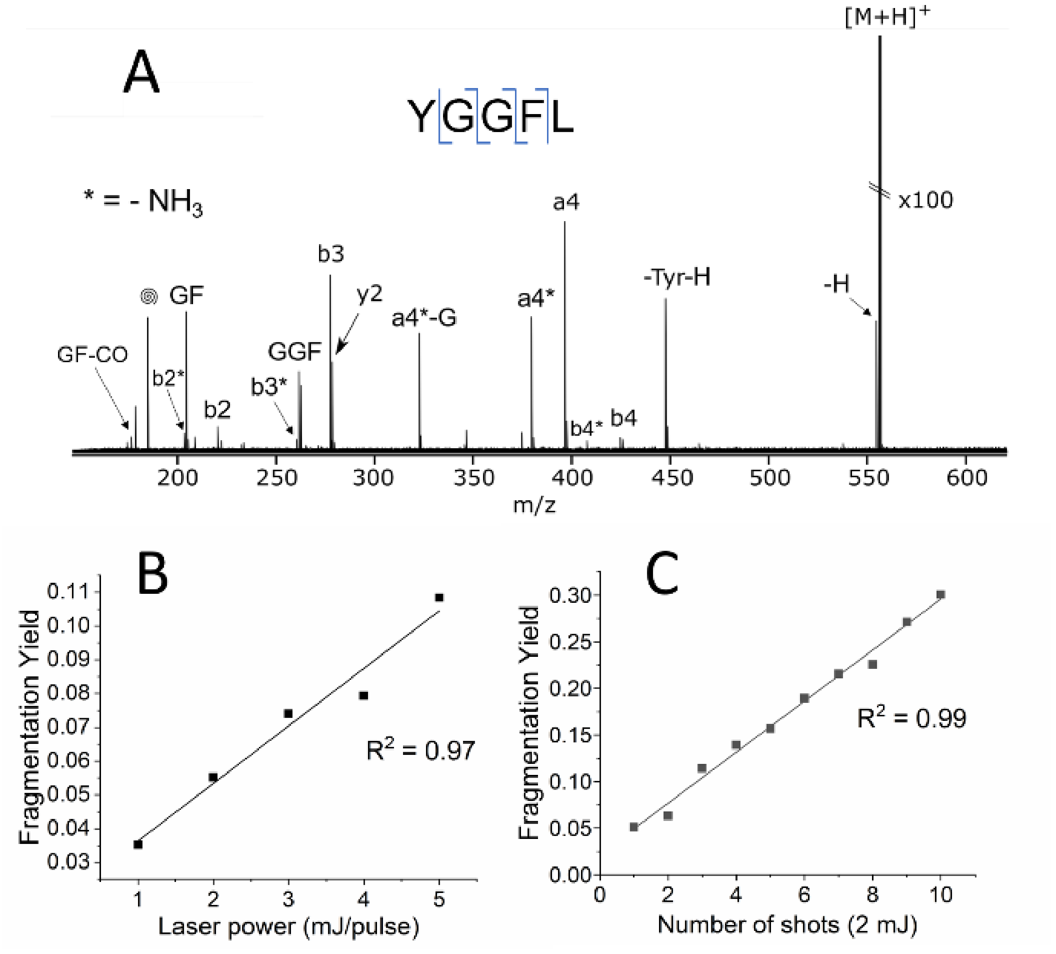

SI Figure 1: 193 nm UVPD of LeuEnk. A) UVPD spectrum taken with 1 laser pulse at 2 mJ pulse energy. B) Fragmentation yield as a function of laser power. C) Fragmentation yield as a function of number of laser shots.

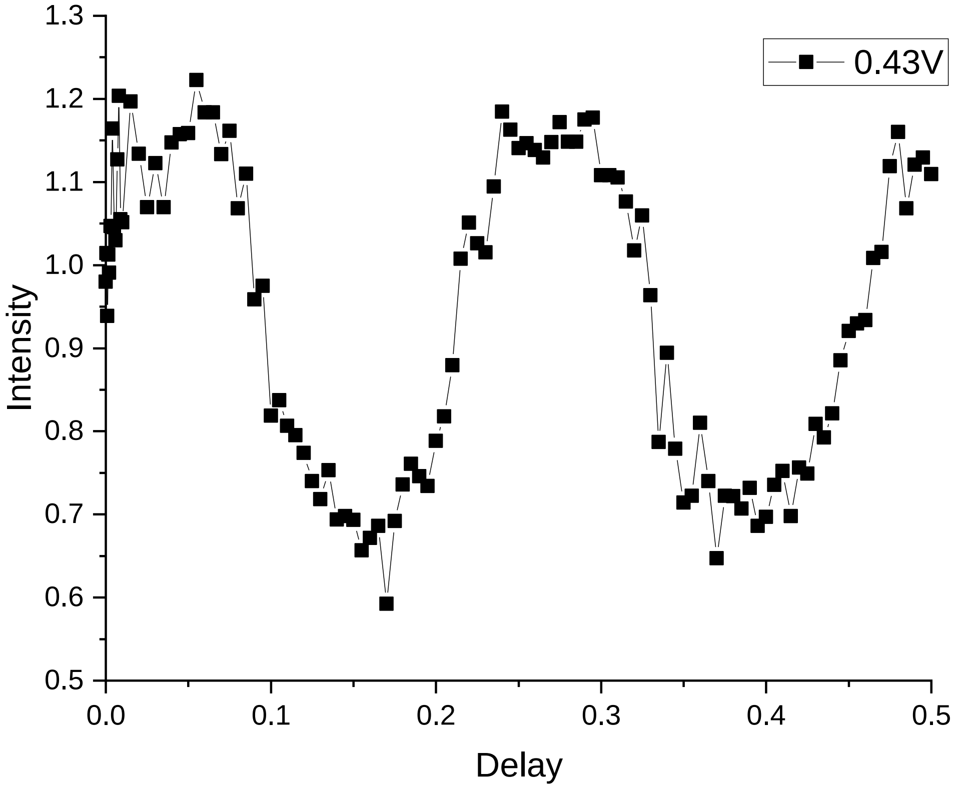

SI Figure 2: Intensity of the 2nd harmonic as a function of the delay between ions entering the cell and the laser being fired. Due to the experimental set up, another 120 ms delay between the laser shot and excitation of the ions existed.

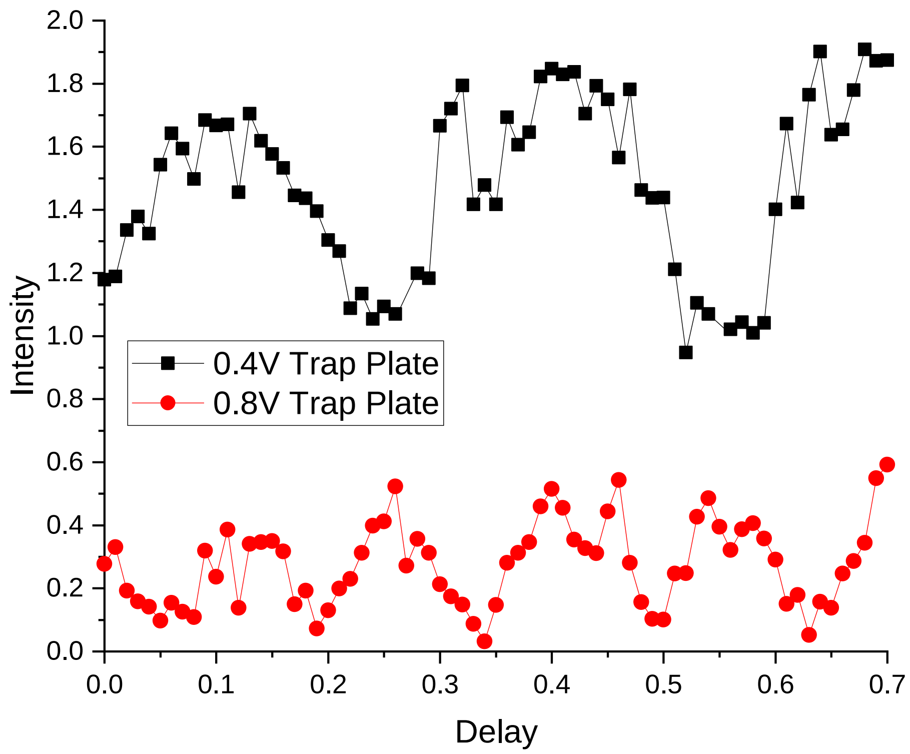

SI Figure 3: Intensity of the 2nd harmonic as a function of UVPD delay compared between low and high trap voltage settings. Doubling the trap voltage doubles magnetron motion frequency.

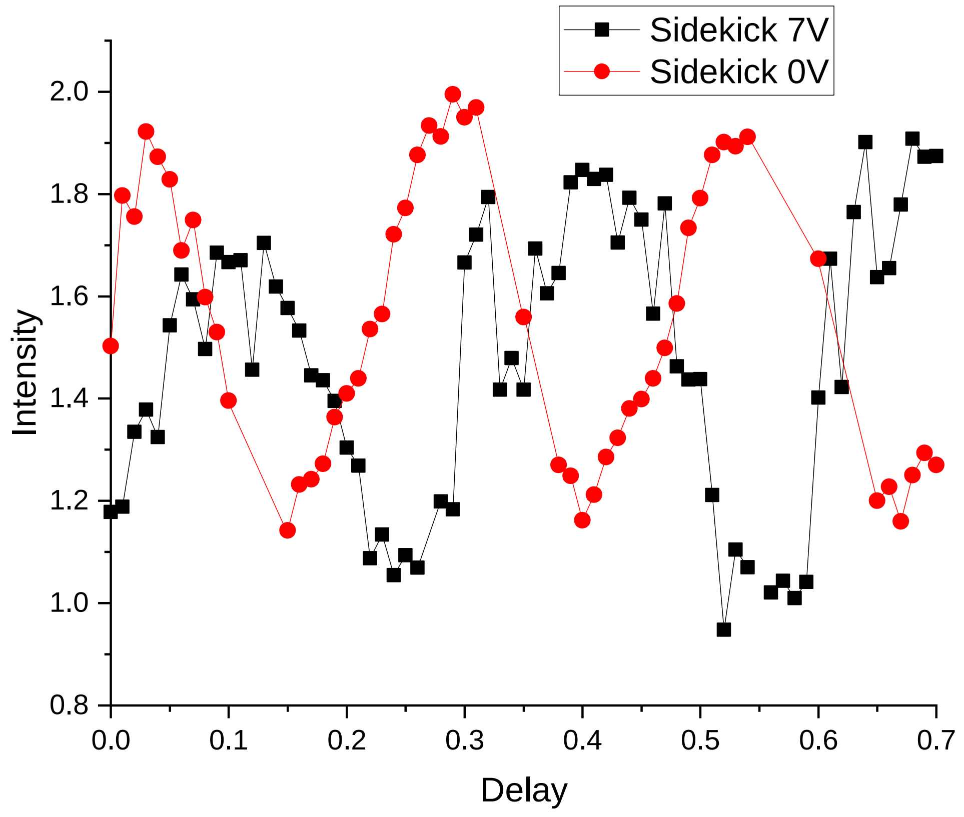

SI Figure 4: Intensity of the 2nd harmonic as a function of UVPD delay compared between a sidekick voltage of 0 V and 7 V, resulting in a different x offset for ions entering the infinity cell.

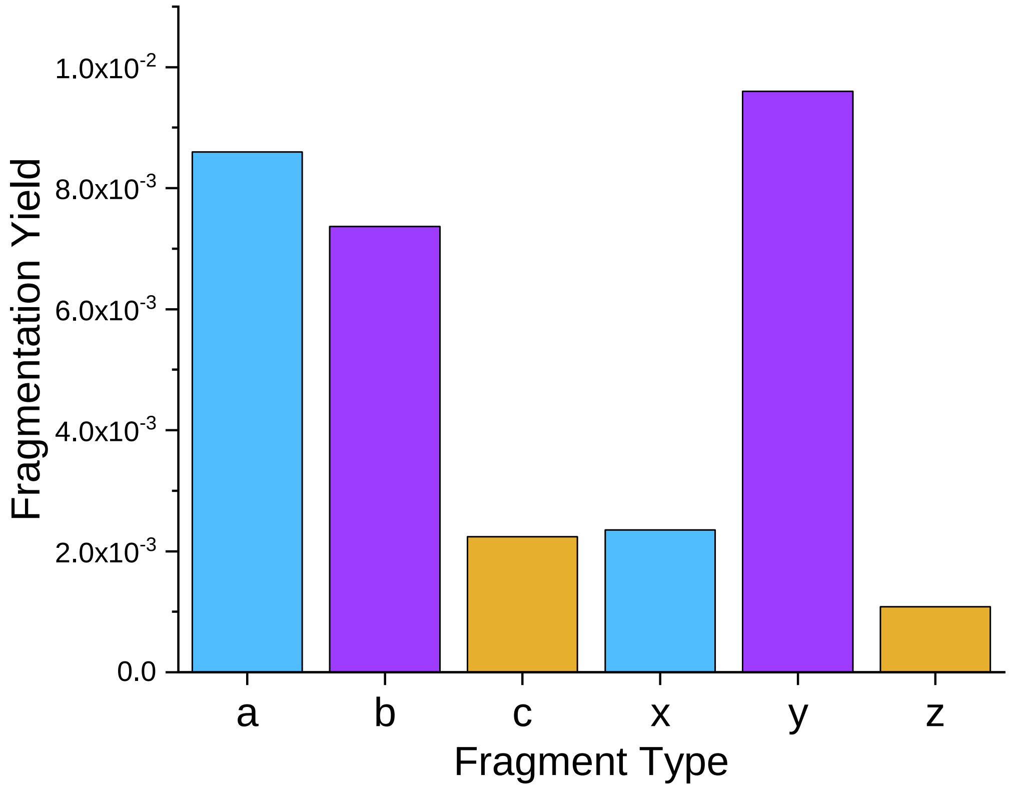

SI Figure 5: Fragmentation yield of 193 nm UVPD of $[M+5H]^{5+}$ by fragment type. a, b and y ions are the most abundant species.

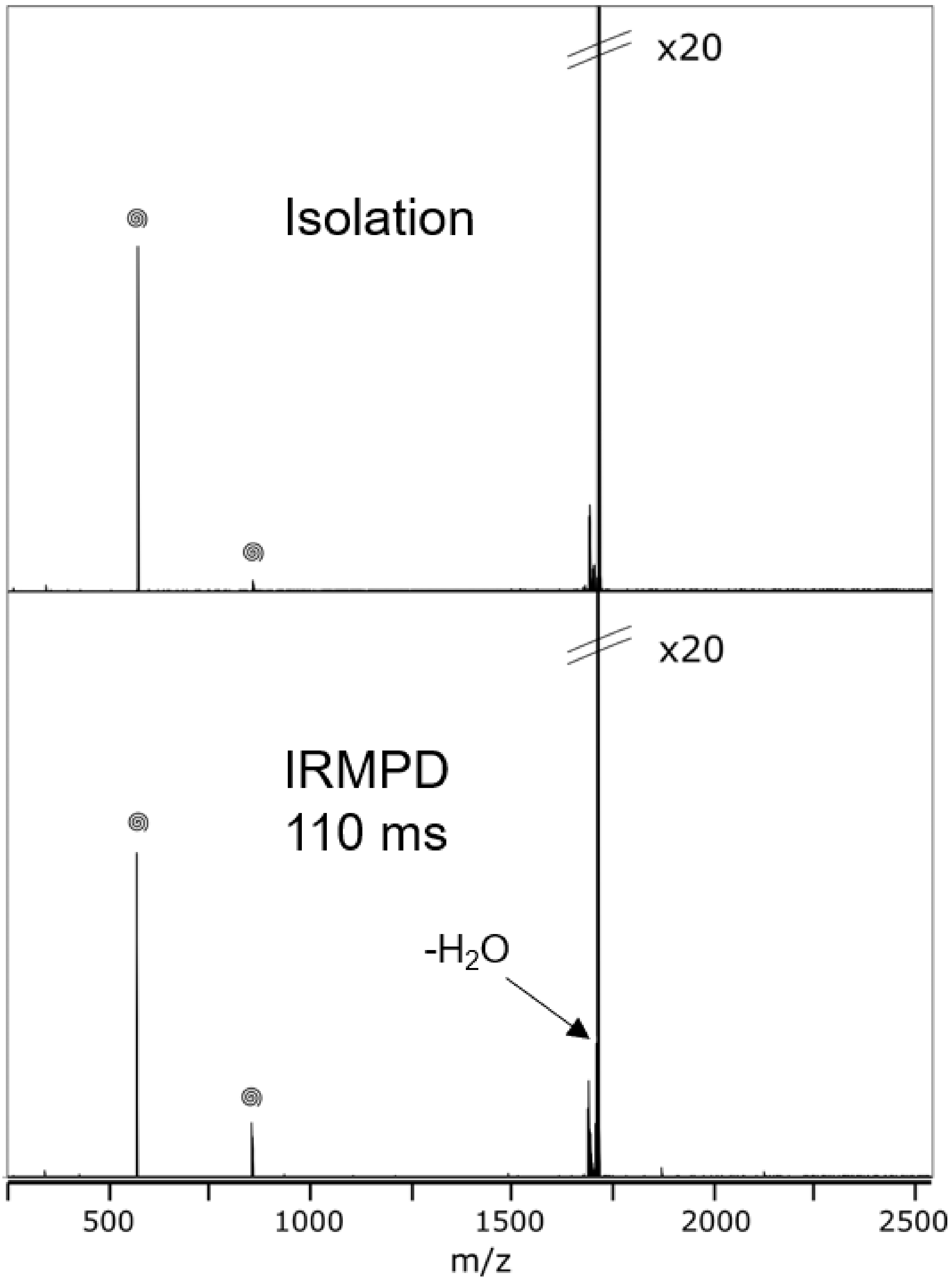

SI Figure 6: Isolation only (top) and IRMPD control spectum (bottom) of $[M+5H]^{5+}$ Ubiquitin. IR irradition of 110 ms, chosen because it is the maximum value at which no significant fragmentation occurs other than small molecule loss, results in loss of water and NH_3 from the precursor ion at ~1% of precursor intensity.

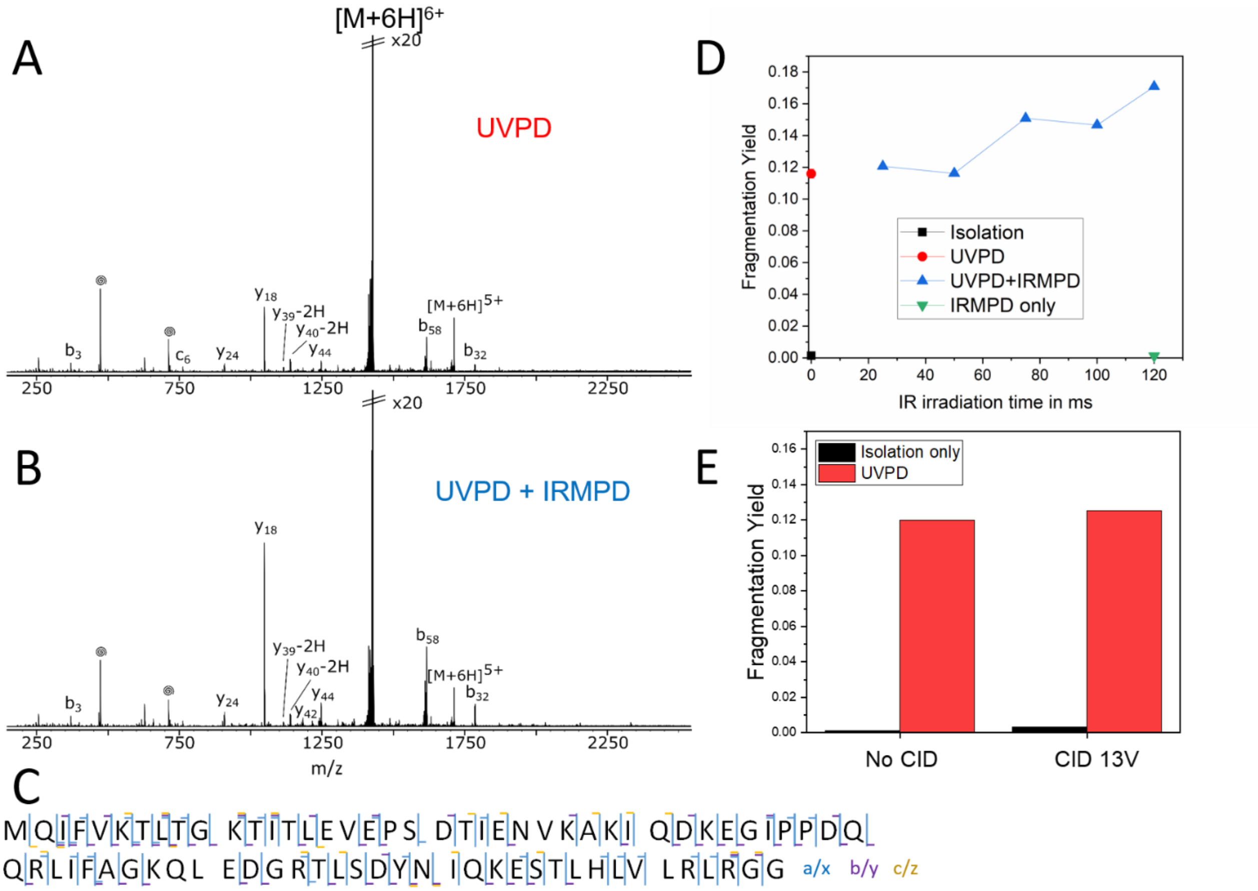

SI Figure 7: 193 nm UVPD of 6+ Ubiquitin with and without subsequent IR activation as well as pre-activation in the collision cell. A) UVPD spectrum obtained with 1 laser pulse at 7 mJ. B) UVPD followed by 120 ms IR irradiation. C) Cleavage coverage map. D) Fragmentation yield as a function of IR irradiation time. E) Fragmentation yield with and without CID prior to UVPD.

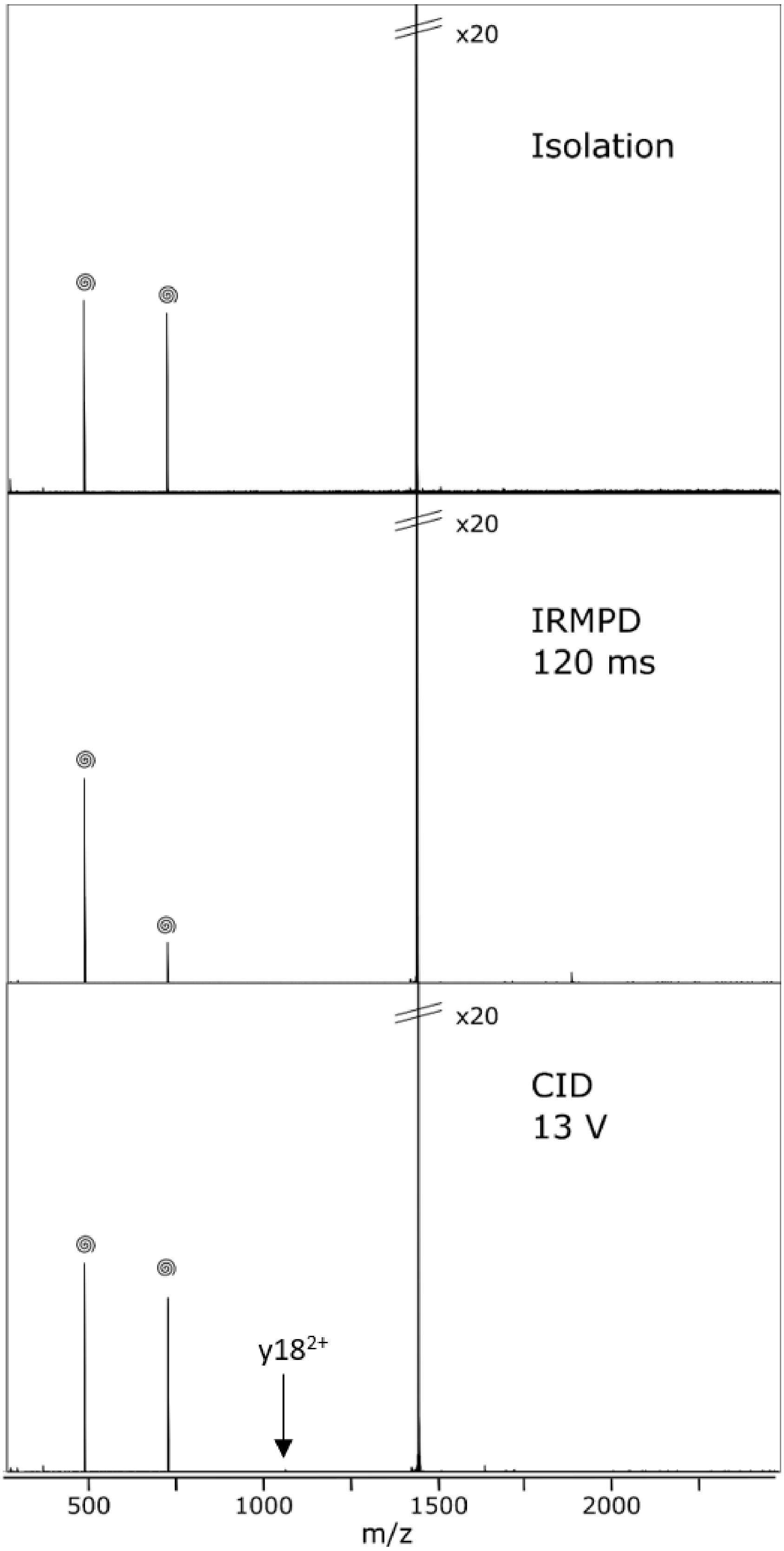

SI Figure 8: Isolation only (top), IRMPD 120 ms (middle) and CID 13 V (bottom) control spectra of $[M+6H]^{6+}$ Ubiquitin. Both fragmentation methods were used at the setting where the first minimal onset of fragmentation was observed. IRMPD showed loss of NH_3 while CID showed loss of NH_3, CO_2 and the sequence fragment $y18^{2+}$.

SI Schematics

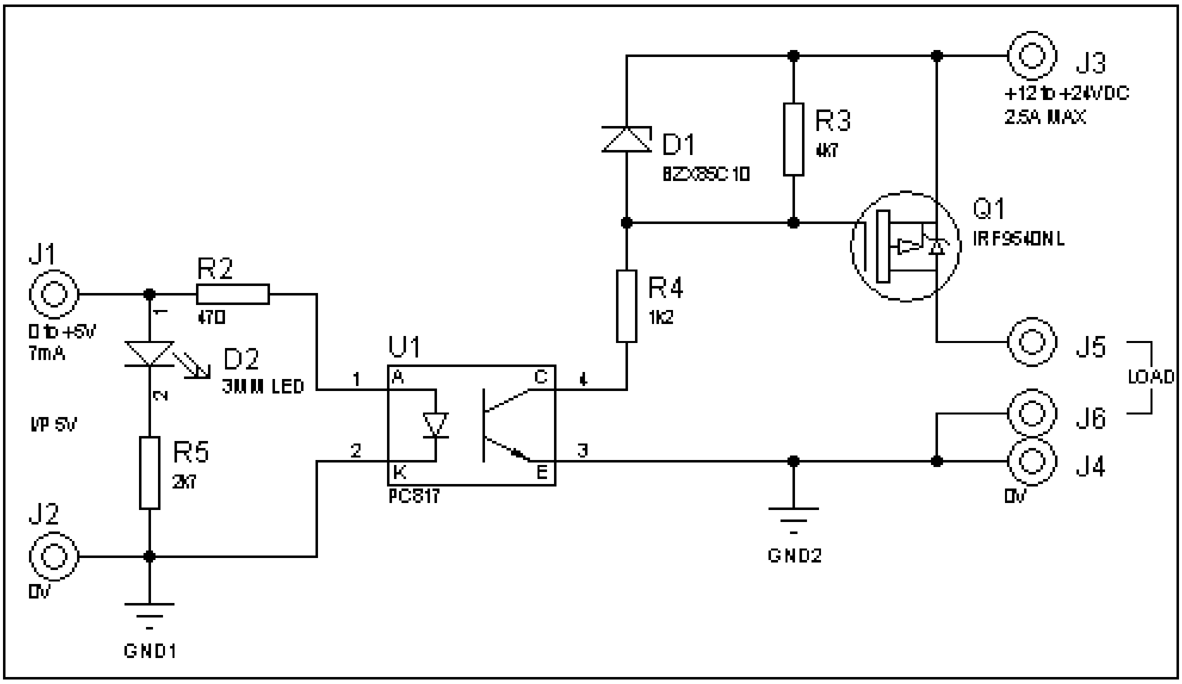

SI schematic 1: Wiring diagram of FET switch used for laser triggering.

SI solariX pulse pograms used in this work

Standard UVPD experiments

```
;===========================================================================
; BASIC : Bruker Acquisition Sequence (for) Instrument Control
;
; A simplified generic event sequence (pulse program) for ftmsControl.
; This version supports ETD and MSn events as well as all other common
; experimental timing, pulse and acquisition events using conditional
; substitution based upon method parameter settings.
;
; Rev 1.0 (??/??/??) -- created for ftmsControl
; Rev 1.1 (02/22/13) -- comment updated for ftmsControl
; Rev 1.2 (03/27/13) -- added POST_CAPTURE_DELAY
; Rev 1.3 (11/23/15) -- added various "PREAMP" events  (for QPD control)
;===========================================================================

;-------------------------------------
;Initialization Block
;-------------------------------------
; INITIALIZE_PREAMP ignored -- not necessary for solariX/Infinity Cell systems
; ~ ~ ~ Excite/Detect Definitions ~ ~ ~
; automatic delays:
; automatic frequency lists:
define list<frequency> ExciteSweep = <ExciteSweep>
; automatic loopcounters:
define loopcounter ExciteSweep_size
; ~ ~ End of Automatic Definitions  ~ ~
```

```
;-------------------------------------
;Hystar autosuspend Block
;-------------------------------------

;-------------------------------------
;Dataset Acquisition Block ("2D" loop)
;-------------------------------------
; AUTO_EXP_DELAY removed for TUNE acquisition
EXP_START,  zd                          ; beginning of each experiment loop, zero buffers

;-------------------------------------
;Scan Accumulation Block (NS loop)
;-------------------------------------
; AUTO_SCAN_DELAY replaced with a delay parameter for DRU Streaming
NS_START, d0                            ; start of scan accumulation (NS) loop
; automatic loopcounters: reset value(s)
"ExciteSweep_size = ExciteSweep.len"
  ; turn on Ultra RF amp (7), set transfer hexapole RF amp=0 (29, no ion transmission)
  1u setnmr4|7 setnmr3|29
; PREAMP_EXCITE_MODE_ENABLE ignored -- not necessary for solariX/Infinity Cell systems
  ; set collision cell Entrance (15) and DC (31) high, to make sure quench works properly
  1u setnmr3|15|31

;-------------------------------------
;Source & Infinity Cell Quench Block
;-------------------------------------
  1u setnmr4|12                               ; source octupole quench on (12)
  25m setnmr3|27 setrtp1|8      ; enable infinity cell quench (27) and collision cell quench (8)
  1u setnmr3^27 setnmr4^12 setrtp1^8 ; all quenches off
  1u setnmr3^29                               ; re-enable transfer hexapole RF (3^29, transmit ions)
```

```
;--------------------------------------
;Dynamic Trap Plate Block (raise)
;--------------------------------------
; gated trapping not enabled

;--------------------------------------
;Ion Accumulation Block (start)
;--------------------------------------
SRCFILL, d1                        ; post quench delay (accumulate in source)

;--------------------------------------
;Accumulation in Collision Cell Block
;--------------------------------------
  1u setnmr3^15^31                 ; set collision cell Entrance and DC for accumulation
  ; pulse train to simultaneously extract ions from source octupole (d10) and open shutter
  ((d10 setnmr3|14 1u setnmr3^14) (1u setnmr3|13))
  d11                              ; post extract delay
; ETD_EXPERIMENT ignored, ETD not enabled

;--------------------------------------
;Accumulation in Infinity Cell Block
;--------------------------------------
  1u setnmr3|15|31                 ; set the collision cell Entrance and DC for ejection
  d2 setnmr3|24 1u setnmr3^24^13       ; transfer ions to infinity cell (24), then close shutter (13)

;--------------------------------------
;Ion Accumulation Block (end)
;(repeat Accumulation blocks for Source, Collision Cell, Infinity Cell)
;--------------------------------------
  lo to SRCFILL times l30; collect L[30] ion generations
```

```
;=====================================================================
; INTERNAL MS/MS
; --> Possible Events automatically generated
;  1) INFINITY_CELL_ISOLATION
;  2) PULSED_VALVE_1, PULSED_VALVE_2 (for post-isolation/pre-SORI)
;  3) SORI
;  4) ECD
;=====================================================================

; --- ISOLATION ---
; IN_CELL_ISOLATION is disabled

; --- PULSED_VALVE(S) ---
; PULSED_VALVE_1 is not enabled for MS/MS
; PULSED_VALVE_2 is not enabled for MS/MS

; --- SORI ---
; SORI not enabled

; --- ECD ---
; ECD not enabled


; --- IRMPD ---
d15
  d25 setnmr4|31                 ; trigger UVPD laser pulse (XGPP_OUT[2])
  1u setnmr4^31


;=====================================================================
```

```
; INTERNAL MS^3
; --> Possible Events automatically generated
;  1) INTERMEDIATE_PUMP_DELAY
;  2) INFINITY_CELL_ISOLATION_MS3
;  3) PULSED_VALVE_1_MS3, PULSED_VALVE_2_MS3 (for post-isolation/pre-SORI)
;  4) SORI_MS3
;  5) ECD_MS3
;======================================================================

; --- INTERMEDIATE_PUMP_DELAY ---
; INTERMEDIATE_PUMP_DELAY not necessary between MSMS and MS^3

; --- ISOLATION ---
; IN_CELL_ISOLATION_MS3 is disabled

; --- PULSED_VALVE(S) ---
; PULSED_VALVE_1 is not enabled for MS^3
; PULSED_VALVE_2 is not enabled for MS^3

; --- SORI ---
; SORI in MS^3 not enabled

; --- ECD ---
; ECD in MS^3 not enabled


;======================================================================
; INTERNAL MS^4
; --> Possible Events automatically generated
;  1) INTERMEDIATE_PUMP_DELAY
;  2) INFINITY_CELL_ISOLATION_MS4
```

```
;  3) PULSED_VALVE_1_MS4, PULSED_VALVE_2_MS4 (for post-isolation/pre-SORI)
;  4) SORI_MS4
;  5) ECD_MS4
;==========================================================================

; --- INTERMEDIATE_PUMP_DELAY ---
; INTERMEDIATE_PUMP_DELAY not necessary between MS^3 and MS^4

; --- ISOLATION ---
; IN_CELL_ISOLATION_MS4 is disabled

; --- PULSED_VALVE(S) ---
; PULSED_VALVE_1 is not enabled for MS^4
; PULSED_VALVE_2 is not enabled for MS^4

; --- SORI ---
; SORI in MS^4 not enabled

; --- ECD ---
; ECD in MS^4 not enabled



;-------------------------------------
;Final pumpdown after all MSn events
;-------------------------------------
; FINAL_PUMP_DELAY not enabled - No ACTIVE PULSED_VALVE events

;-------------------------------------
;Dynamic Trap Plate Block (lower)
;-------------------------------------
; gated trapping not enabled
```

```
;--------------------------------------
;Post Capture Delay (PCD)
;--------------------------------------
; POST_CAPTURE_DELAY ignored -- not necessary for solariX/Infinity Cell systems



;==========================================================================
;        EXCITATION AND DETECTION
;==========================================================================

; reset phase immediately before Excitation event
   10u reset:f1                           ; reset phase of DDS in FCtrl-1


;--------------------------------------
;Excitation Block
;--------------------------------------
   10u pl3:f1                             ; set attenuation for excitation (FCtrl-1)
EXC_SWP, p3 ph1 ExciteSweep:f1 ExciteSweep.inc          ; broadcast current list entry and increment pointer
   lo to EXC_SWP times ExciteSweep_size            ; ExciteSweep_size entries/steps in excite list


;--------------------------------------
;Detection Block
;--------------------------------------
   ; turn off Ultra RF amp before detect (7), set transfer hexapole RF amp=0 (29, no ion transmission)
   1u setnmr4^7 setnmr3|29
; PREAMP_DETECT_MODE_ENABLE ignored -- not necessary for solariX/Infinity Cell systems
   go = NS_START ph1                  ; scan accumulation (loop to NS_START times NS)


;--------------------------------------
```

```
;Write, Serial Loop, Exit Block
;-------------------------------------
  10u wr #0                       ; write data to disk
  exit                            ; end acquisition/experiment


;-------------------------------------
; Phase program definitions for FCUs
;-------------------------------------
  ph1= 0 2 0 2                    ; phase program: 0 180 0 180 (exc/det RF for 'standard' (1w) mode)
  ph2= 0 0 0 0                    ; phase program: 0  0  0  0  (all other 'general' RF events)
  ph3= 0 2 0 2                    ; phase program: 0 180 0 180 (excitation RF for QPD (2w) mode)
  ph4= 0 0 0 0                    ; phase program: 0  0  0  0  (detection RF for QPD (2w) mode)


;=====================================================================
; This pulse program was generated using:
; -> EventKeyNameDefs_Bruker.properties
; -> IPSO_Bruker.properties
; -> MS3_MS4_Bruker.properties
;=====================================================================
```

UVPD followed by IRMPD experiments

```
;=========================================================================
; BASIC : Bruker Acquisition Sequence (for) Instrument Control
;
; A simplified generic event sequence (pulse program) for ftmsControl.
; This version supports ETD and MSn events as well as all other common
; experimental timing, pulse and acquisition events using conditional
; substitution based upon method parameter settings.
```

```
;
; Rev 1.0 (??/??/??) -- created for ftmsControl
; Rev 1.1 (02/22/13) -- comment updated for ftmsControl
; Rev 1.2 (03/27/13) -- added POST_CAPTURE_DELAY
; Rev 1.3 (11/23/15) -- added various "PREAMP" events  (for QPD control)
;==============================================================================

;-------------------------------------
;Initialization Block
;-------------------------------------
; INITIALIZE_PREAMP ignored -- not necessary for solariX/Infinity Cell systems
; ~ ~ ~ Excite/Detect Definitions ~ ~ ~
; automatic delays:
; automatic frequency lists:
define list<frequency> ExciteSweep = <ExciteSweep>
; automatic loopcounters:
define loopcounter ExciteSweep_size
; ~ ~ End of Automatic Definitions  ~ ~

;-------------------------------------
;Hystar autosuspend Block
;-------------------------------------

;-------------------------------------
;Dataset Acquisition Block ("2D" loop)
;-------------------------------------
; AUTO_EXP_DELAY removed for TUNE acquisition
EXP_START,  zd                          ; beginning of each experiment loop, zero buffers

;-------------------------------------
;Scan Accumulation Block (NS loop)
```

```
;--------------------------------------
; AUTO_SCAN_DELAY replaced with a delay parameter for DRU Streaming
NS_START, d0                    ; start of scan accumulation (NS) loop
; automatic loopcounters: reset value(s)
"ExciteSweep_size = ExciteSweep.len"
  ; turn on Ultra RF amp (7), set transfer hexapole RF amp=0 (29, no ion transmission)
  1u setnmr4|7 setnmr3|29
; PREAMP_EXCITE_MODE_ENABLE ignored -- not necessary for solariX/Infinity Cell systems
  ; set collision cell Entrance (15) and DC (31) high, to make sure quench works properly
  1u setnmr3|15|31

;--------------------------------------
;Source & Infinity Cell Quench Block
;--------------------------------------
  1u setnmr4|12                          ; source octupole quench on (12)
  25m setnmr3|27 setrtp1|8     ; enable infinity cell quench (27) and collision cell quench (8)
  1u setnmr3^27 setnmr4^12 setrtp1^8 ; all quenches off
  1u setnmr3^29                          ; re-enable transfer hexapole RF (3^29, transmit ions)

;--------------------------------------
;Dynamic Trap Plate Block (raise)
;--------------------------------------
; gated trapping not enabled

;--------------------------------------
;Ion Accumulation Block (start)
;--------------------------------------
SRCFILL, d1                     ; post quench delay (accumulate in source)

;--------------------------------------
;Accumulation in Collision Cell Block
```

```
;-------------------------------------
   1u setnmr3^15^31              ; set collision cell Entrance and DC for accumulation
   ; pulse train to simultaneously extract ions from source octupole (d10) and open shutter
   ((d10 setnmr3|14 1u setnmr3^14) (1u setnmr3|13))
   d11                           ; post extract delay
; ETD_EXPERIMENT ignored, ETD not enabled

;-------------------------------------
;Accumulation in Infinity Cell Block
;-------------------------------------
   1u setnmr3|15|31              ; set the collision cell Entrance and DC for ejection
   d2 setnmr3|24 1u setnmr3^24^13      ; transfer ions to infinity cell (24), then close shutter (13)

;-------------------------------------
;Ion Accumulation Block (end)
;(repeat Accumulation blocks for Source, Collision Cell, Infinity Cell)
;-------------------------------------
   lo to SRCFILL times l30; collect L[30] ion generations

;=====================================================================
; INTERNAL MS/MS
; --> Possible Events automatically generated
;  1) INFINITY_CELL_ISOLATION
;  2) PULSED_VALVE_1, PULSED_VALVE_2 (for post-isolation/pre-SORI)
;  3) SORI
;  4) ECD
;=====================================================================

; --- ISOLATION ---
; IN_CELL_ISOLATION is disabled
```

```
; --- PULSED_VALVE(S) ---
; PULSED_VALVE_1 is not enabled for MS/MS
; PULSED_VALVE_2 is not enabled for MS/MS

; --- SORI ---
; SORI not enabled

; --- ECD ---
; ECD not enabled


; --- IRMPD ---
d15
  d25 setnmr4|31                    ; trigger UVPD laser pulse (XGPP_OUT[2])
  1u setnmr4^31
d17
  d13 setnmr3|12                    ; trigger IRMPD laser pulse (XGPP_OUT[2])
  10u setnmr3^12

;==================================================================
; INTERNAL MS^3
; --> Possible Events automatically generated
;  1) INTERMEDIATE_PUMP_DELAY
;  2) INFINITY_CELL_ISOLATION_MS3
;  3) PULSED_VALVE_1_MS3, PULSED_VALVE_2_MS3 (for post-isolation/pre-SORI)
;  4) SORI_MS3
;  5) ECD_MS3
;==================================================================

; --- INTERMEDIATE_PUMP_DELAY ---
; INTERMEDIATE_PUMP_DELAY not necessary between MSMS and MS^3
```

```
; --- ISOLATION ---
; IN_CELL_ISOLATION_MS3 is disabled

; --- PULSED_VALVE(S) ---
; PULSED_VALVE_1 is not enabled for MS^3
; PULSED_VALVE_2 is not enabled for MS^3

; --- SORI ---
; SORI in MS^3 not enabled

; --- ECD ---
; ECD in MS^3 not enabled


;============================================================================
; INTERNAL MS^4
; --> Possible Events automatically generated
;  1) INTERMEDIATE_PUMP_DELAY
;  2) INFINITY_CELL_ISOLATION_MS4
;  3) PULSED_VALVE_1_MS4, PULSED_VALVE_2_MS4 (for post-isolation/pre-SORI)
;  4) SORI_MS4
;  5) ECD_MS4
;============================================================================

; --- INTERMEDIATE_PUMP_DELAY ---
; INTERMEDIATE_PUMP_DELAY not necessary between MS^3 and MS^4

; --- ISOLATION ---
; IN_CELL_ISOLATION_MS4 is disabled
```

; --- PULSED_VALVE(S) ---

; PULSED_VALVE_1 is not enabled for MS^4

; PULSED_VALVE_2 is not enabled for MS^4

; --- SORI ---

; SORI in MS^4 not enabled

; --- ECD ---

; ECD in MS^4 not enabled

;--------------------------------------

;Final pumpdown after all MSn events

;--------------------------------------

; FINAL_PUMP_DELAY not enabled - No ACTIVE PULSED_VALVE events

;--------------------------------------

;Dynamic Trap Plate Block (lower)

;--------------------------------------

; gated trapping not enabled

;--------------------------------------

;Post Capture Delay (PCD)

;--------------------------------------

; POST_CAPTURE_DELAY ignored -- not necessary for solariX/Infinity Cell systems

;===

; EXCITATION AND DETECTION

;===

```
; reset phase immediately before Excitation event
  10u reset:f1                    ; reset phase of DDS in FCtrl-1


;-------------------------------------
;Excitation Block
;-------------------------------------
  10u pl3:f1                      ; set attenuation for excitation (FCtrl-1)
EXC_SWP, p3 ph1 ExciteSweep:f1 ExciteSweep.inc        ; broadcast current list entry and increment pointer
  lo to EXC_SWP times ExciteSweep_size          ; ExciteSweep_size entries/steps in excite list


;-------------------------------------
;Detection Block
;-------------------------------------
  ; turn off Ultra RF amp before detect (7), set transfer hexapole RF amp=0 (29, no ion transmission)
  1u setnmr4^7 setnmr3|29
; PREAMP_DETECT_MODE_ENABLE ignored -- not necessary for solariX/Infinity Cell systems
  go = NS_START ph1               ; scan accumulation (loop to NS_START times NS)


;-------------------------------------
;Write, Serial Loop, Exit Block
;-------------------------------------
  10u wr #0                       ; write data to disk
  exit                            ; end acquisition/experiment


;-------------------------------------
; Phase program definitions for FCUs
;-------------------------------------
  ph1= 0 2 0 2                    ; phase program: 0 180 0 180 (exc/det RF for 'standard' (1w) mode)
  ph2= 0 0 0 0                    ; phase program: 0  0  0  0  (all other 'general' RF events)
  ph3= 0 2 0 2                    ; phase program: 0 180 0 180 (excitation RF for QPD (2w) mode)
```

```
  ph4= 0 0 0 0                          ; phase program: 0  0  0  0  (detection RF for QPD (2w) mode)



;======================================================================

; This pulse program was generated using:

; -> EventKeyNameDefs_Bruker.properties

; -> IPSO_Bruker.properties

; -> MS3_MS4_Bruker.properties

;======================================================================
```

UVPD-2DMS experiments, default properties

```
#=====================================================================

# IPSO console specific pulse program definitions.

# For systems with "NICE" (Nifty Intelligent Control Electronics)

#=====================================================================

# $Revision: 1.24 $

#=====================================================================


#--------------------------------------------------------------------

# These definitions are used by ftmsControl to generate a pulse

# program from BASIC for goFTMS.

#

# Note that the event names MUST match the definitions for the

# corresponding event key in EventKeyNameFile

#---------------------------------------------------------------------


#---------------------------------------------------------------------

# event definitions are listed alphabetically

#---------------------------------------------------------------------
```

COLL_CELL_ACCUM_KEY:

Note 1: there are unique ESI and MALDI versions

Note 2: <SHUTTER_DELAY> will be replaced with a absolute delay during generation of pulse program

COLL_CELL_ACCUM_ESI.lines = 4

COLL_CELL_ACCUM_ESI.1 = "   1u setnmr3^15^31   ; set collision cell Entrance and DC for accumulation"

COLL_CELL_ACCUM_ESI.2 = "   ; pulse train to simultaneously extract ions from source octupole (d10) and open shutter"

COLL_CELL_ACCUM_ESI.3 = "   ((d10 setnmr3|14 1u setnmr3^14) (<SHUTTER_DELAY> setnmr3|13))"

COLL_CELL_ACCUM_ESI.4 = "   d11   ; post extract delay"

COLL_CELL_ACCUM_ESI_CASI.lines = 10

COLL_CELL_ACCUM_ESI_CASI.1 = "   1u setnmr3^15^31   ; set collision cell Entrance and DC for accumulation"

COLL_CELL_ACCUM_ESI_CASI.2 = "   ;open shutter and extract ions from source"

COLL_CELL_ACCUM_ESI_CASI.3 = "   10u setnmr3|13"

COLL_CELL_ACCUM_ESI_CASI.4 = "   1u setnmr3|14"

COLL_CELL_ACCUM_ESI_CASI.5 = "   d10   ;   for time slice 0 only";

COLL_CELL_ACCUM_ESI_CASI.6 = "   ISOLATION_LOOP, 1u   ;   loop number of isolation windows-1"

COLL_CELL_ACCUM_ESI_CASI.7 = "   300u setnmr3|4 1u setnmr3^4   ; advance to next time slice"

COLL_CELL_ACCUM_ESI_CASI.8 = "   d10   ;   for current time slice";

COLL_CELL_ACCUM_ESI_CASI.9 = "   lo to ISOLATION_LOOP times <NUMBER_OF_ISOLATION>   ; Do exp on other isolation window -1"

COLL_CELL_ACCUM_ESI_CASI.10 = "   d11 setnmr3^14   ; post extract delay"

COLL_CELL_ACCUM_MALDI.lines = 7

COLL_CELL_ACCUM_MALDI.1 = "   ; simultaneously open shutter, set source octupole for extract, collision cell dc for accumulation"

COLL_CELL_ACCUM_MALDI.2 = "   <SHUTTER_DELAY> setnmr3|13|14^15^31   ; add minimum delay required for shutter"

COLL_CELL_ACCUM_MALDI.3 = "   LASER_SHOTS, d28   ; set repetition rate for laser"

COLL_CELL_ACCUM_MALDI.4 = "   10u setnmr3|11   ; Laser ON"

COLL_CELL_ACCUM_MALDI.5 = "   1u setnmr3^11   ; Laser OFF"

COLL_CELL_ACCUM_MALDI.6 = "   lo to LASER_SHOTS times l28 ; L[28] laser shots"

COLL_CELL_ACCUM_MALDI.7 = "   d11 setnmr3^14   ; turn off source octupole extract during post-extract delay"

COLL_CELL_ACCUM_MALDI_MTP2.lines = 6

COLL_CELL_ACCUM_MALDI_MTP2.1 = "   ; simultaneously open shutter, set source octupole for extract, collision cell dc for accumulation"

COLL_CELL_ACCUM_MALDI_MTP2.2 = "   <SHUTTER_DELAY> setnmr3|13|14^15^31   ; add minimum delay required for shutter"

COLL_CELL_ACCUM_MALDI_MTP2.3 = "   10u trignl4   ; check for level on MTPBusy to be low"

COLL_CELL_ACCUM_MALDI_MTP2.4 = "   60u setnmr3|11 1u setnmr3^11   ; trigger MTP2 laser sequence"

COLL_CELL_ACCUM_MALDI_MTP2.5 = "   10u trigne4   ; wait for signal from MTP2 (laser sequence finished)"

COLL_CELL_ACCUM_MALDI_MTP2.6 = "   d11 setnmr3^14   ; turn off source octupole extract during post-extract delay"

COLL_CELL_ACCUM_MALDI_MTP2_CASI.lines = 9

COLL_CELL_ACCUM_MALDI_MTP2_CASI.1 = "   ; simultaneously open shutter, set source octupole for extract, collision cell dc for accumulation"

COLL_CELL_ACCUM_MALDI_MTP2_CASI.2 = "   1u setnmr3|13|14^15^31   ; add minimum delay required for shutter"

COLL_CELL_ACCUM_MALDI_MTP2_CASI.3 = "   LASER_TRIGGER, 1u   ; "

COLL_CELL_ACCUM_MALDI_MTP2_CASI.4 = "   10u trignl4   ; check for level on MTPBusy to be low"

COLL_CELL_ACCUM_MALDI_MTP2_CASI.5 = "   60u setnmr3|11 1u setnmr3^11   ; trigger MTP2 laser sequence"

COLL_CELL_ACCUM_MALDI_MTP2_CASI.6 = "   10u trigne4   ; wait for signal from MTP2 (laser sequence finished)"

COLL_CELL_ACCUM_MALDI_MTP2_CASI.7 = "   300u setnmr3|4 1u setnmr3^4 ; advance to next time slice"

COLL_CELL_ACCUM_MALDI_MTP2_CASI.8 = "   lo to LASER_TRIGGER times <NUM_ISOLATION>   ; Do exp on other isolations"

COLL_CELL_ACCUM_MALDI_MTP2_CASI.9 = "   d11 setnmr3^14   ; turn off source octupole extract during post-extract delay"

COLL_CELL_DC_HIGH_KEY:

COLL_CELL_DC_HIGH.lines = 2

COLL_CELL_DC_HIGH.1 = "   ; set collision cell Entrance (15) and DC (31) high, to make sure quench works properly"

COLL_CELL_DC_HIGH.2 = "   1u setnmr3|15|31"

COLL_CELL_QUENCH_KEY: (part of SOURCE_QUENCH event)

COLL_CELL_QUENCH.lines = 3

COLL_CELL_QUENCH.1 = "   25m setrtp1|8   ; collision cell quench on"

COLL_CELL_QUENCH.2 = "   1u setrtp1^8   ; collision cell quench off"

COLL_CELL_QUENCH.3 = "   1u setnmr4^12   ; source octupole quench off"

CORRELATED_SHOTS_KEY:

CORRELATED_SHOTS.lines = 3

CORRELATED_SHOTS.1 = "   10u pl7:f1   ; set attenuation for correlated shots (FCtrl-1)"

CORRELATED_SHOTS.2 = "CR_SHOTS, p7 ph2 <CORR_SHOTS_LIST>:f1 <CORR_SHOTS_LIST>.inc   ; correlated shots"

CORRELATED_SHOTS.3 = "   lo to CR_SHOTS times <CORR_SHOTS_LIST>_size   ; <CORR_SHOTS_LIST>_size shots"

CORRELATED_SWEEP_KEY:

CORRELATED_SWEEP.lines = 3

CORRELATED_SWEEP.1 = "   10u pl4:f1   ; set attenuation for correlated sweep (FCtrl-1)"

CORRELATED_SWEEP.2 = "CR_SWP, p4 ph2 <CORR_SWEEP_LIST>:f1 <CORR_SWEEP_LIST>.inc
            ; correlated sweep"

CORRELATED_SWEEP.3 = "   lo to CR_SWP times <CORR_SWEEP_LIST>_size   ; <CORR_SWEEP_LIST>_size steps in sweep"

DETECTION_KEY:

DETECTION.lines = 1

DETECTION.1 = "   go = NS_START <DETECT_PHASE_PROGRAM>   ; scan accumulation (loop to NS_START times NS)"

DYNAMIC_TRAP_LOWER_KEY:

Note: This is now a "wrapper key" and no longer has a corresponding definition.

It will automatically be replaced one of the events:

DYNAMIC_TRAP_LOWER_AUTOMATIC or DYNAMIC_TRAP_LOWER_TRIGGERED

based on the Gated_Trapping_Mode parameter

DYNAMIC_TRAP_LOWER_AUTOMATIC_KEY:

DYNAMIC_TRAP_LOWER_AUTOMATIC.lines = 1

#DYNAMIC_TRAP_LOWER_AUTOMATIC.1 = "   <DYN_TRAP_TIME> setnmr3|20 1u setnmr3^20
; send pulse to trigger start of automatic voltage ramp"

use a pulse train for automatic mode

DYNAMIC_TRAP_LOWER_AUTOMATIC.1 = "   ((<DYN_TRAP_TIME>) (10u setnmr3|20 1u setnmr3^20))   ; send pulse to trigger start of automatic voltage ramp"

DYNAMIC_TRAP_LOWER_TRIGGERED_KEY:

DYNAMIC_TRAP_LOWER_TRIGGERED.lines = 3

DYNAMIC_TRAP_LOWER_TRIGGERED.1 = "   DT_LOW, <DYN_TRAP_TIME> setnmr3|20   ; lower dynamic trap voltage with voltage ramp"

DYNAMIC_TRAP_LOWER_TRIGGERED.2 = "   1u setnmr3^20"

DYNAMIC_TRAP_LOWER_TRIGGERED.3 = "   lo to DT_LOW times <DYN_TRAP_STEPS>   ; loop for each step in voltage ramp"

DYNAMIC_TRAP_RAISE_KEY:

DYNAMIC_TRAP_RAISE.lines = 1

DYNAMIC_TRAP_RAISE.1 = "   10u setnmr3|20 1u setnmr3^20   ; send pulse to trigger initial voltage of ramp"

ECD_KEY:

ECD.lines = 2

ECD.1 = "   d12 setnmr3|26   ; ECD pulse (INT_GATE)"

ECD.2 = "   10u setnmr3^26"

ETD_EXPERIMENT_KEY: Note: line 21 is dynamic for ETD lock mass injection

ETD_EXPERIMENT.lines = 23

ETD_EXPERIMENT.1 = "   ; ---------- ETD BLOCK ----------"

ETD_EXPERIMENT.2 = "   <ETD_SKIP>   ;   skip unnecessary time slices"

#ETD_EXPERIMENT.2 = "   SKIP_SLICE, 1u   ;   skip number of isolation windows"

#ETD_EXPERIMENT.3 = "   200u setnmr3|4 1u setnmr3^4   ; skip/advance to next time slice"

#ETD_EXPERIMENT.4 = "   lo to SKIP_SLICE times <SKIP_NUMBER>   ; skip unnecessary time slices"

ETD_EXPERIMENT.3 = "   1u setnmr4|24   ; turn on ion mirror"

ETD_EXPERIMENT.4 = "   10m   ; delay with ion mirror and dc on"

ETD_EXPERIMENT.5 = "   300u setnmr3|4 1u setnmr3^4 ; advance to time slice-14(1) set reagent inject potentials"

ETD_EXPERIMENT.6 = "   300u setnmr3|4   ; advance to time slice-15(2) set reagent trans. potentials"

ETD_EXPERIMENT.7 = "   10m   ; settling of potentials for reagent transmission"

ETD_EXPERIMENT.8 = "   1u setnmr3^4"

ETD_EXPERIMENT.9 = "   300u setnmr3|4|30   ; advance to time slice-16(3) open nci gate"

ETD_EXPERIMENT.10 = "   d23   ; reagent accumulation"

ETD_EXPERIMENT.11 = "   1u setnmr3^4^30"

ETD_EXPERIMENT.12 = "   1m"

ETD_EXPERIMENT.13 = "   300u setnmr3|4   ; advance to time slice-17(4) set potentials for reaction"

ETD_EXPERIMENT.14 = "   d20   ; reaction"

ETD_EXPERIMENT.15 = "   1u setnmr3^4"

ETD_EXPERIMENT.16 = "   300u setnmr3|4 1u setnmr3^4   ; advance to time slice-18(5) reagent quench"

ETD_EXPERIMENT.17 = "   1u setnmr4^24   ; turn off ion mirror"

ETD_EXPERIMENT.18 = "   d11   ; post extract delay"

ETD_EXPERIMENT.19 = "   <ETD_LOCK_MASS>   ; go to time slice-19(6) for lock mass"

```
ETD_EXPERIMENT.20 = "   300u setnmr3|4                    ; reset to time slice-0"

ETD_EXPERIMENT.21 = "   d11                               ; post extract delay"

ETD_EXPERIMENT.22 = "   1u  setnmr3^4"

ETD_EXPERIMENT.23 = "   ; ---------- ETD BLOCK ----------"



# EXCITATION_KEY:

# Note: This is now a "wrapper key" and no longer has a corresponding definition.

#       It uses one of the events:

#           EXCITATION_SHAPE, EXCITATION_SHOT or EXCITATION_SWEEP

#       based on the EM parameter


# EXCITATION_SHAPE_KEY:

EXCITATION_SHAPE.lines = 1

EXCITATION_SHAPE.1 = "   (p0:sp0 <EXCITE_PHASE_PROGRAM>):f1                    ; broadcast
excitation shaped pulse on FCtrl-1"


# EXCITATION_SHOT_KEY:

EXCITATION_SHOT.lines = 2

EXCITATION_SHOT.1 = "   10u pl3:f1                            ; set attenuation for excitation (FCtrl-1)"

EXCITATION_SHOT.2 = "EXC_SHOT, p3 <EXCITE_PHASE_PROGRAM> <EXCITE_SHOT>:f1   ; detection
excitation shot"


# EXCITATION_SWEEP_KEY:

EXCITATION_SWEEP.lines = 12

EXCITATION_SWEEP.1 = "           10u pl27:f1                             ; set attenuation for excitation
(FCtrl-1)"

EXCITATION_SWEEP.2 = "EXC_SWPA, p27 ph1 ExciteSweep:f1 ExciteSweep.inc    ; broadcast current
list entry and increment pointer"

EXCITATION_SWEEP.3 = "lo to EXC_SWPA times ExciteSweep_size           ;
<EXCITE_SWEEP_LIST>_size entries/steps in excite list"

EXCITATION_SWEEP.4 = "d26 id26"

EXCITATION_SWEEP.5 = "10u pl27:f1                         ; set attenuation for excitation (FCtrl-1)"
```

EXCITATION_SWEEP.6 = "EXC_SWPB, p27 ph1 ExciteSweep:f1 ExciteSweep.inc ; broadcast current list entry and increment pointer"

EXCITATION_SWEEP.7 = "lo to EXC_SWPB times ExciteSweep_size ; <EXCITE_SWEEP_LIST>_size entries/steps in excite list"

EXCITATION_SWEEP.8 = "d25 setnmr4|31 ; UVPD pulse (INT_GATE)"

EXCITATION_SWEEP.9 = "1u setnmr4^31"

EXCITATION_SWEEP.10 = "10u pl3:f1 ; set attenuation for excitation (FCtrl-1)"

EXCITATION_SWEEP.11 = "EXC_SWP, p3 ph1 ExciteSweep:f1 ExciteSweep.inc ; broadcast current list entry and increment pointer"

EXCITATION_SWEEP.12 = "lo to EXC_SWP times ExciteSweep_size ; <EXCITE_SWEEP_LIST>_size entries/steps in excite list"

EXCITATION_SWEEP_RAMPED_POWER_KEY:

EXCITATION_SWEEP_RAMPED_POWER.lines = 3

EXCITATION_SWEEP_RAMPED_POWER.1 = "   10u <EXCITE_SWEEP_LIST>[0]:f1 ; set attenuation for excitation (FCtrl-1)"

EXCITATION_SWEEP_RAMPED_POWER.2 = "EXC_SWP, p3 <EXCITE_PHASE_PROGRAM> <EXCITE_SWEEP_LIST>:f1 <EXCITE_SWEEP_LIST>.inc ExcitePower:f1 ExcitePower.inc ; broadcast current list entry and increment pointer"

EXCITATION_SWEEP_RAMPED_POWER.3 = "   lo to EXC_SWP times <EXCITE_SWEEP_LIST>_size ; <EXCITE_SWEEP_LIST>_size entries/steps in excite list"

EXP_START_KEY:

Note: <AUTO_EXP_DELAY> will be replaced with a absolute delay during generation of pulse program

EXP_START.lines = 1

EXP_START.1 = "EXP_START, <AUTO_EXP_DELAY> zd ; beginning of each experiment loop, zero buffers"

FCU_RESET_KEY:

FCU_RESET.lines = 1

FCU_RESET.1 = "   10u reset:f1 ; reset phase of DDS in FCtrl-1"

FINAL_PUMP_DELAY_KEY:

```
FINAL_PUMP_DELAY.lines = 1

FINAL_PUMP_DELAY.1 = "   3s                              ; final pumpdown delay"


# FREQUENCY_SWEEP_1_KEY:

FREQUENCY_SWEEP_1.lines = 3

FREQUENCY_SWEEP_1.1 = "   10u pl5:f1                          ; set attenuation for frequency sweep 1 (FCtrl-1)"

FREQUENCY_SWEEP_1.2 = "FREQ_SWP_1, p5 ph2 <FREQ_SWEEP_LIST>:f1 <FREQ_SWEEP_LIST>.inc             ; frequency sweep 1"

FREQUENCY_SWEEP_1.3 = "   lo to FREQ_SWP_1 times <FREQ_SWEEP_LIST>_size            ; <FREQ_SWEEP_LIST>_size steps in sweep"


# FREQUENCY_SWEEP_2_KEY:

FREQUENCY_SWEEP_2.lines = 3

FREQUENCY_SWEEP_2.1 = "   10u pl6:f1                          ; set attenuation for frequency sweep 2 (FCtrl-1)"

FREQUENCY_SWEEP_2.2 = "FREQ_SWP_2, p6 ph2 <FREQ_SWEEP_LIST>:f1 <FREQ_SWEEP_LIST>.inc             ; frequency sweep 2"

FREQUENCY_SWEEP_2.3 = "   lo to FREQ_SWP_2 times <FREQ_SWEEP_LIST>_size            ; <FREQ_SWEEP_LIST>_size steps in sweep"


# FREQUENCY_SWEEP_3_KEY:

FREQUENCY_SWEEP_3.lines = 3

FREQUENCY_SWEEP_3.1 = "   10u pl10:f1                         ; set attenuation for frequency sweep 3 (FCtrl-1)"

FREQUENCY_SWEEP_3.2 = "FREQ_SWP_3, p10 ph2 <FREQ_SWEEP_LIST>:f1 <FREQ_SWEEP_LIST>.inc             ; frequency sweep 3"

FREQUENCY_SWEEP_3.3 = "   lo to FREQ_SWP_3 times <FREQ_SWEEP_LIST>_size            ; <FREQ_SWEEP_LIST>_size steps in sweep"


# FREQUENCY_SWEEP_4_KEY:

FREQUENCY_SWEEP_4.lines = 3

FREQUENCY_SWEEP_4.1 = "   10u pl11:f1                         ; set attenuation for frequency sweep 4 (FCtrl-1)"
```

FREQUENCY_SWEEP_4.2 = "FREQ_SWP_4, p11 ph2 <FREQ_SWEEP_LIST>:f1 <FREQ_SWEEP_LIST>.inc ; frequency sweep 4"

FREQUENCY_SWEEP_4.3 = " lo to FREQ_SWP_4 times <FREQ_SWEEP_LIST>_size ; <FREQ_SWEEP_LIST>_size steps in sweep"

HYSTAR_AUTOSUSPEND_KEY:

HYSTAR_AUTOSUSPEND.lines = 1

HYSTAR_AUTOSUSPEND.1 = " 100u autosuspend ; wait for Hystar"

INFINITY_CELL_ACCUM EVENT:

Note 1: there are unique ESI and MALDI versions

Note 2: currently both ESI and MALDI use identical definitions

INFINITY_CELL_ACCUM_ESI.lines = 2

INFINITY_CELL_ACCUM_ESI.1 = " 1u setnmr3|15|31 ; set the collision cell Entrance and DC for ejection"

INFINITY_CELL_ACCUM_ESI.2 = " d2 setnmr3|24 1u setnmr3^24^13 ; transfer ions to infinity cell (24), then close shutter (13)"

INFINITY_CELL_ACCUM_MALDI.lines = 2

INFINITY_CELL_ACCUM_MALDI.1 = " 1u setnmr3|15|31 ; set the collision cell Entrance and DC for ejection"

INFINITY_CELL_ACCUM_MALDI.2 = " d2 setnmr3|24 1u setnmr3^24^13 ; transfer ions to infinity cell (24), then close shutter (13)"

Note : For multi CASI, both ESI and MALDI use identical definitions

INFINITY_CELL_ACCUM_CASI.lines = 5

INFINITY_CELL_ACCUM_CASI.1 = " 1u setnmr3|15|31 ; set the collision cell Entrance and DC for ejection"

INFINITY_CELL_ACCUM_CASI.2 = " d2 setnmr3|24 1u setnmr3^24^13 ; transfer ions to infinity cell (24), then close shutter (13)"

INFINITY_CELL_ACCUM_CASI.3 = " SKIP_SLICE, 1u ; skip number of isolation windows"

INFINITY_CELL_ACCUM_CASI.4 = " 200u setnmr3|4 1u setnmr3^4 ; advance to next time slice"

INFINITY_CELL_ACCUM_CASI.5 = " lo to SKIP_SLICE times <SKIP_NUMBER> ; skip unnecessary time slices"

INFINITY_CELL_ISOLATION_KEY:

Note: This is a "wrapper key" and does not have a corresponding definition. It uses the

CORRELATED_SHOTS and CORRELATED_SWEEP events based on IN_CELL_ISOLATION_MODE parameter

INFINITY_CELL_QUENCH_KEY:

INFINITY_CELL_QUENCH.lines = 2

INFINITY_CELL_QUENCH.1 = "   25m setnmr3|27   ; infinity cell quench (QUENCH)"

INFINITY_CELL_QUENCH.2 = "   1u setnmr3^27"

INITIALIZE_PREAMP_KEY:

Note 1: there are 2 different preamp modes, each with 2 sub-mode settings that are selected at run/compile time

so we use a dynamic event here that is properly replaced during event sequence to pulse program parsing

Note 2: this event definition is critical to the safety of the preamp. Using the wrong setting can DESTROY the

preamp and is therefore NOT user definable.

INITIALIZE_PREAMP.lines = 1

INITIALIZE_PREAMP.1 = "<INITIALIZE_PREAMP>"

ION_QUENCH_KEY: (combined quench event for SOURCE_QUENCH and INFINITY_CELL_QUENCH)

Note that for optimal quench on solariX systems:

1) the transfer hexapole RF should be disabled DURING the CC and infinity cell quench

2) we assume (1) is done prior to this event in the pulse program (presumably done in ULTRA_RF_ON)

3) and then re-enable transfer hexapole RF for ion transmission AFTER the quenches (3^29)

ION_QUENCH.lines = 3

ION_QUENCH.1 = "   25m setnmr3|27 setrtp1|8 ; enable infinity cell quench (27) and collision cell quench (8)"

ION_QUENCH.2 = "   1u setnmr3^27 setnmr4^12 setrtp1^8 ; all quenches off"

ION_QUENCH.3 = "   1u setnmr3^29   ; re-enable transfer hexapole RF (3^29, transmit ions)"

MALDI_RANDOM_WALK_KEY:

MALDI_RANDOM_WALK.lines = 2

MALDI_RANDOM_WALK.1 = "   10u setnmr3|18   ; Trigger for random walk"

MALDI_RANDOM_WALK.2 = "   1u setnmr3^18"

NEXT_VAR_DELAY_KEY:

NEXT_VAR_DELAY.lines = 1

NEXT_VAR_DELAY.1 = "   ivd   ; increment to next delay in list"

NEXT_VAR_PULSE_KEY:

NEXT_VAR_PULSE.lines = 1

NEXT_VAR_PULSE.1 = "   ivp   ; increment to next pulse in list"

PHASE_PROGRAM_DEFS_KEY:

PHASE_PROGRAM_DEFS.lines = 4

PHASE_PROGRAM_DEFS.1 = "   ph1= 0 2 0 2   ; phase program: 0 180 0 180 (exc/det RF for 'standard' (1w) mode)"

PHASE_PROGRAM_DEFS.2 = "   ph2= 0 0 0 0   ; phase program: 0 0 0 0 (all other 'general' RF events)"

PHASE_PROGRAM_DEFS.3 = "   ph3= 0 2 0 2   ; phase program: 0 180 0 180 (excitation RF for QPD (2w) mode)"

PHASE_PROGRAM_DEFS.4 = "   ph4= 0 0 0 0   ; phase program: 0 0 0 0 (detection RF for QPD (2w) mode)"

POST_CAPTURE_DELAY_KEY:

POST_CAPTURE_DELAY.lines = 1

POST_CAPTURE_DELAY.1 = "   d24   ; Post Capture Delay (PCD) for Para Cell"

PREAMP_DETECT_MODE_ENABLE_KEY:

Note: this event definition is critical to the safety of the preamp. Using the wrong setting can DESTROY the

preamp and is therefore NOT user definable. The specific commands appropriate for the analyzer cell

(Para Cell vs. Infinity Cell) will be provided during pulse program compilation.

PREAMP_DETECT_MODE_ENABLE.lines = 1

PREAMP_DETECT_MODE_ENABLE.1 = "<PREAMP_DETECT_MODE_ENABLE>"

PREAMP_EXCITE_MODE_ENABLE_KEY:

Note: this event definition is critical to the safety of the preamp. Using the wrong setting can DESTROY the

preamp and is therefore NOT user definable. The specific commands appropriate for the analyzer cell

(Para Cell vs. Infinity Cell) will be provided during pulse program compilation.

PREAMP_EXCITE_MODE_ENABLE.lines = 1

PREAMP_EXCITE_MODE_ENABLE.1 = "<PREAMP_EXCITE_MODE_ENABLE>"

PULSED_VALVE_1_KEY:

PULSED_VALVE_1.lines = 2

PULSED_VALVE_1.1 = "   5m setnmr3|22   ; Pulsed Valve 1 (VALVE1)"

PULSED_VALVE_1.2 = "   1u setnmr3^22"

PULSED_VALVE_2_KEY:

PULSED_VALVE_2.lines = 2

PULSED_VALVE_2.1 = "   5m setnmr3|23   ; Pulsed Valve 2 (VALVE2)"

PULSED_VALVE_2.2 = "   1u setnmr3^23"

PUMP_DELAY_KEY:

PUMP_DELAY.lines = 1

PUMP_DELAY.1 = "   d6   ; pumping delay"

REPETITIVE_ION_ACCUM_KEY:

REPETITIVE_ION_ACCUM.lines = 1

REPETITIVE_ION_ACCUM.1 = "   lo to SRCFILL times l30   ; collect L[30] ion generations"

SCAN_START_KEY:

Note 1: <AUTO_SCAN_DELAY> will be replaced with a absolute delay during generation of pulse program

Note 2: <AUTO_LOOPCOUNTER_INIT> will be replaced with automatic loopcounter initialization events

except for ADD acquisitions, when these events must be located inside the INF_CELL_ACCUM loop

SCAN_START.lines = 2

SCAN_START.1 = "NS_START, <AUTO_SCAN_DELAY>   ; start of scan accumulation (NS) loop"

SCAN_START.2 = "<AUTO_LOOPCOUNTER_INIT>"

SHAPED_PULSE_KEY:

SHAPED_PULSE.lines = 1

SHAPED_PULSE.1 = "   (p1:sp1 ph1):f1   ; broadcast shaped pulse on FCtrl-1"

SORI_KEY:

SORI.lines = 2

SORI.1 = "   10u pl8:f1   ; set attenuation for activation (FCtrl-1)"

SORI.2 = "   p8 ph2 <SORI_FREQ>:f1   ; activation shot on the parent ion"

SOURCE_ACCUM_KEY:

SOURCE_ACCUM.lines = 1

SOURCE_ACCUM.1 = "SRCFILL, d1   ; post quench delay (accumulate in source)"

SOURCE_HEX_QUENCH_KEY: (part of SOURCE_QUENCH event)

SOURCE_HEX_QUENCH.lines = 1

SOURCE_HEX_QUENCH.1 = "   1u setnmr4|12   ; source octupole quench on (12)"

TIMESLICE_ADVANCE_PULSE_KEY:

TIMESLICE_ADVANCE_PULSE.lines = 2

TIMESLICE_ADVANCE_PULSE.1 = " 300u setnmr3|4   ; Set TTL high to advance table"

TIMESLICE_ADVANCE_PULSE.2 = " 1u setnmr3^4"

ULTRA_RF_OFF_KEY:

Note that when the Ultra RF is disabled (e.g. for ion detection) we also need to disable

the transfer hexapole RF (3|29) to minimize noise bleed.

ULTRA_RF_OFF.lines = 2

ULTRA_RF_OFF.1 = "   ; turn off Ultra RF amp before detect (7), set transfer hexapole RF amp=0 (29, no ion transmission)"

ULTRA_RF_OFF.2 = "   1u setnmr4^7 setnmr3|29"

ULTRA_RF_ON_KEY:

Note that when the Ultra RF is enabled (presumably only at beginning of each scan, after SCAN_START),

we also want to disable the transfer hexapole RF (3|29) to optimize ion quenching (no ion transfer to cell).

ULTRA_RF_ON.lines = 2

ULTRA_RF_ON.1 = "   ; turn on Ultra RF amp (7), set transfer hexapole RF amp=0 (29, no ion transmission)"

ULTRA_RF_ON.2 = "   1u setnmr4|7 setnmr3|29"

USER_EVENT_1_KEY:

USER_EVENT_1.lines = 0

USER_EVENT_2_KEY:

USER_EVENT_2.lines = 0

USER_EVENT_3_KEY:

USER_EVENT_3.lines = 0

```
# USER_EVENT_4_KEY:
USER_EVENT_4.lines = 0

# USER_EVENT_5_KEY:
USER_EVENT_5.lines = 0

# USER_EVENT_6_KEY:
USER_EVENT_6.lines = 0

# USER_EVENT_7_KEY:
USER_EVENT_7.lines = 0

# USER_EVENT_8_KEY:
USER_EVENT_8.lines = 0

# USER_EVENT_9_KEY:
USER_EVENT_9.lines = 0

# USER_EVENT_10_KEY:
USER_EVENT_10.lines = 0

# VAR_DELAY_KEY:
VAR_DELAY.lines = 1
VAR_DELAY.1 = "   vd                              ; variable delay"

# VAR_PULSE_KEY:
VAR_PULSE.lines = 1
VAR_PULSE.1 = "   vp                              ; variable pulse"

# WRITE_DATA_KEY:
# Note: This is a "wrapper key" and does not have a corresponding definition.
```

```
#       It uses one of the events:
#          WRITE_DATA_1D, WRITE_DATA_2D, WRITE_DATA_2D_ADD or
WRITE_DATA_PARAM_CHANGE
#       based on the LC_mode, IonAccumulationDuringDelay and IonChargeControl parameters

# Single Fid (1D):
WRITE_DATA_1D.lines = 2
WRITE_DATA_1D.1 = "   10u wr #0                 ; write data to disk"
WRITE_DATA_1D.2 = "   exit                      ; end acquisition/experiment"

# Serial (2D):
WRITE_DATA_2D.lines = 4
WRITE_DATA_2D.1 = "   10u wr #0                 ; write data to disk"
WRITE_DATA_2D.2 = "   50u autosuspend"
WRITE_DATA_2D.3 = "   lo to EXP_START times l20"
WRITE_DATA_2D.4 = "   exit                ; end acquisition/experiment"

# Serial (2D) with ADD:
WRITE_DATA_2D_ADD.lines = 5
WRITE_DATA_2D_ADD.1 = "   10u wr #0            ; write data to disk"
WRITE_DATA_2D_ADD.2 = "   50u autosuspend      ; wait for 'resume' from ftmsControl"
WRITE_DATA_2D_ADD.3 = "   10u zd               ; zero memory buffers in DRU for next scan"
WRITE_DATA_2D_ADD.4 = "   lo to INF_CELL_ACCUM times <MAX_2D_DATASETS>      ; acquire a
maximum of <MAX_2D_SCANS> datasets"
WRITE_DATA_2D_ADD.5 = "   exit                       ; end acquisition/experiment"

# Serial (2D) with parameter change capability
WRITE_DATA_PARAM_CHANGE.lines = 6
WRITE_DATA_PARAM_CHANGE.1 = "   d30 wr #0                   ; get data from DRU, delay
(TBD) for d10 upload"
WRITE_DATA_PARAM_CHANGE.2 = "   resume_calc          ; resume duration generator,
continue experiment"
```

WRITE_DATA_PARAM_CHANGE.3 = "   d31   ; delay (TBD) for DSP to reload FIFO"

WRITE_DATA_PARAM_CHANGE.4 = "   stop_calc   ; stop duration generator - allow for d10 change"

WRITE_DATA_PARAM_CHANGE.5 = "   lo to EXP_START times <MAX_2D_DATASETS>   ; acquire a maximum of <MAX_2D_SCANS> datasets"

WRITE_DATA_PARAM_CHANGE.6 = "   exit   ; end acquisition/experiment"

2DMS – UVPD experiments, master properties

#==

master configuration properties for pulse program generation

#

IMPORTANT: DO NOT use '.' in filenames! Please use '_' or '-'

#==

$Revision: 1.3 $

#==

#--

Console specific event definition files

#

Format is:

Console type = console specific property file name

#

Note that the Console type must match the configured console

type defined in .../config/hardware.properties

Possible values: currently only "IPSO" supported

#--

IPSO   = 2D_UVPD_default

#--

Internal MS^3 and MS^4 specific event definitions

```
#-----------------------------------------------------------------

InternalMSnFile= MS3_MS4_Bruker


#-----------------------------------------------------------------

# Event Key --> Event Name definition file

#-----------------------------------------------------------------

EventKeyNameFile = EventKeyNameDefs_Bruker


#-----------------------------------------------------------------

# Accumulation During Detection (ADD) event key:name definition file

# NOTE: only used if "Ion Accumulation During Detection" is enabled.

#-----------------------------------------------------------------

ADD_EventKeyNameFile = ADD_EventKeyNameDefs_Bruker


#-----------------------------------------------------------------

# Accumulation During Detection (ADD) event definition file

# NOTE: only used if "Ion Accumulation During Detection" is enabled.

#-----------------------------------------------------------------

ADD_EventsFile = ADD_Bruker


#-----------------------------------------------------------------

# Post Capture Delay (PCD) Optimize event key:name definition file

# NOTE: only used if "PCD_Optimize_Mode" is enabled.

#-----------------------------------------------------------------

PCD_Optimize_EventKeyNameFile = PCD_Optimize_EventKeyNameDefs_Bruker


#-----------------------------------------------------------------

# Post Capture Delay (PCD) Optimize event definition file

# NOTE: only used if "PCD_Optimize_Mode" is enabled.

#-----------------------------------------------------------------

PCD_Optimize_EventsFile = PCD_Optimize_Bruker
```

SI Programs

Cookson 3.0 Fragment analysis and laser control software

The programs written in-house can be found on Github:

https://github.com/CWootton1/Cookson3.0andUVPD
